# *RNA-on-X 1* and *2* in *Drosophila melanogaster* fulfill separate functions in dosage compensation

**DOI:** 10.1371/journal.pgen.1007842

**Published:** 2018-12-10

**Authors:** Maria Kim, Marie-Line Faucillion, Jan Larsson

**Affiliations:** Department of Molecular Biology, Umeå University, Umeå, Sweden; Brown University, UNITED STATES

## Abstract

In *Drosophila melanogaster*, the male-specific lethal (MSL) complex plays a key role in dosage compensation by stimulating expression of male X-chromosome genes. It consists of MSL proteins and two long noncoding RNAs, *roX1* and *roX2*, that are required for spreading of the complex on the chromosome and are redundant in the sense that loss of either does not affect male viability. However, despite rapid evolution, both *roX* species are present in diverse Drosophilidae species, raising doubts about their full functional redundancy. Thus, we have investigated consequences of deleting *roX1* and/or *roX2* to probe their specific roles and redundancies in *D*. *melanogaster*. We have created a new mutant allele of *roX2* and show that *roX1* and *roX2* have partly separable functions in dosage compensation. In larvae, *roX1* is the most abundant variant and the only variant present in the MSL complex when the complex is transmitted (physically associated with the X-chromosome) in mitosis. Loss of *roX1* results in reduced expression of the genes on the X-chromosome, while loss of *roX2* leads to MSL-independent upregulation of genes with male-biased testis-specific transcription. In *roX1 roX2* mutant, gene expression is strongly reduced in a manner that is not related to proximity to high-affinity sites. Our results suggest that high tolerance of mis-expression of the X-chromosome has evolved. We propose that this may be a common property of sex-chromosomes, that dosage compensation is a stochastic process and its precision for each individual gene is regulated by the density of high-affinity sites in the locus.

## Author summary

In humans and fruit flies, females and males have different sets of sex chromosomes. This causes gene dosage differences that must be compensated for by adjusting the expression of most genes located on the X-chromosome. Long non-coding RNAs are central in this compensation and in fruit flies this is mediated by two non-coding RNAs, *roX1* and *roX2* which together with five proteins form the male-specific lethal complex. The complex recognizes and upregulates gene transcription on the male X-chromosome. While non-coding RNAs are are engaged in numerous biological processes and critical for compensation their precise functions remain elusive. To understand the function of long non-coding RNAs we analysed the expression of all genes in *roX1*, *roX2* and *roX1 roX2* mutants to explore the roles of long non-coding RNAs. These mutants have different impacts on the genome-wide expression. Our results also suggest that the X-chromosome is highly tolerant to mis-expression and we speculate that this tolerance evolved in parallel with compensation mechanisms and may be a common property of sex-chromosomes. We propose that dosage compensation is a stochastic process that depends on the distribution of specific binding sites which will be selected for and optimized depending on the genes’ individual expression levels.

## Introduction

In eukaryotic genomes several long non-coding RNAs (lncRNAs) are associated with chromatin and involved in gene expression regulation, but the mechanisms involved are largely unknown. In both mammals and fruit flies, they are required to specifically identify and mark X-chromosomes for dosage compensation, a mechanism that helps maintain balanced expression of the genome. The evolution of sex-chromosomes, for example the X and Y chromosome pairs found in mammals and flies, leads to between-gender differences in gene dosage. Although some genes located on the X-chromosome are expressed in a sex-specific mode, equal expression of most of the genes in males and females is required [1, 2]. Thus, gradual degeneration of the proto-Y chromosome causes an increasing requirement to equalize gene expression between a single X in males and two X-chromosomes in females. X-chromosome expression must also be balanced with expression of the two sets of autosomal chromosomes. Several fundamentally different mechanisms that solve the gene dosage problem and provide such balance have evolved [1–4]. In mammals, one of the pair of X-chromosomes in females is largely silenced through random X-chromosome inactivation, a mechanism that involves at least three lncRNAs [5, 6]. One, the long noncoding *Xist* RNA, plays a key role in marking one of the X-chromosomes and recruiting Polycomb repressive complex 2, thereby mediating its inactivation by histone H3 lysine 27 methylation [7].

In fruit flies, the gene dosage problem has been solved in an apparently opposite way, as X-chromosomal gene expression is increased by approximately a factor of two in males [2, 3]. This increase is mediated by a combination of general buffering effects that act on all monosomic regions [8–10] and the specific targeting and stimulation of the male X-chromosome by the male-specific lethal (MSL) complex. The MSL complex consists of at least five protein components (MSL1, MSL2, MSL3, MLE, and MOF) and two lncRNAs, *roX1* and *roX2* [3, 11, 12]. Although the mammalian and fly compensatory systems respectively inactivate and activate chromosomes in members of different sexes, both rely on lncRNA for correct targeting. Results of UV-mediated crosslinking analyses suggest that only one species of *roX* is present per MSL complex in *Drosophila* [13]. Furthermore, inclusion of a *roX* species is essential for maintaining correct targeting of the MSL complex to the X-chromosome [14]. Upregulation of the male X-chromosome is considered to be partly due to enrichment of histone 4 lysine 16 acetylation (H4K16ac), mediated by the acetyltransferase MOF. The increased expression of X-linked genes in male flies is generally accepted, but the mechanisms involved have not been elucidated. Proposed mechanisms, which are hotly debated [15–17], include increased transcriptional initiation [18, 19], increased elongation [20, 21] or an inverse dosage effect [22].

The *roX1* and *roX2* RNAs differ in sequence and size (3.7 kb versus 0.6 kb) but can still individually support assembly of a functional MSL complex. In an early study of *roX1* and *roX2*, a short homologous stretch was detected [23], which subsequently led to the definition of conserved regions shared by the two RNAs named roX-boxes, located in their 3’ ends [24–26]. Confirmatory genetic studies have shown that expression of six tandem repeats of a 72-bp stem loop region from *roX2* is sufficient for mediation of the MSL complex’s X-chromosome binding and initiation of H4-Lys16 acetylation in the absence of endogenous *roX* RNA [24].

The *roX* RNAs are not maternally deposited and transcription of *roX1* is initiated in both male and female embryos at the beginning of the blastoderm stage [27]. Females subsequently lose *roX1* expression and a few hours after *roX1* is first detected *roX2* appears, but only in males [28].

Despite differences in size, sequence and initial expression, the two *roX* RNAs are functionally redundant in the sense that mutations of either *roX1* or *roX2* alone do not affect male viability and they both co-localize with the MSL complex along the male X-chromosome [23, 27]. In contrast, double (*roX1 roX2*) mutations, which cause a systematic redistribution of the MSL complex, are lethal for most males [29–32]. It should be noted that in *roX1 roX2* mutant the reduction in MSL complex abundance on the male X-chromosome is dramatic; more pronounced than the reductions observed in *mle* or *mof* mutants [14]. Nevertheless, some *roX1 roX2* mutant males may survive, while *mle*, *msl1*, *msl2*, *msl3* or *mof* loss-of-function mutations are completely male-lethal [29–31]. Whether other RNA species can fulfill the role of *roX* RNAs in these instances or the MSL complex can function without RNA species remains to be clarified. Furthermore, the degree of lethality in *roX1 roX2* mutant is highly sensitive to several modifying factors, such as expression levels of MSL1 and MSL2 [33], expression of hairpin RNAs [34, 35], presence and parental source of the Y-chromosome [31], and a functional siRNA pathway [36]. The observations that *roX1 roX2* mutations are not completely lethal and there are several modifying factors suggest an additional layer of redundancy in the role of lncRNAs in chromosome-specific targeting.

To further our understanding of the role of lncRNAs (particularly specific roles and redundancies of *roX1* and *roX2*) in chromosome-specific regulation we here provide a comprehensive expression analysis of *roX1*, *roX2* and *roX1 roX2* mutants to explore the redundancy as well as the differences between the two lncRNA species. We show that *roX1* and *roX2* have partly separable functions in dosage compensation. In larvae, *roX1* is the most abundant variant and the only variant present in the MSL complex when the complex is transmitted (physically associated with the X-chromosome) in mitosis. Loss of *roX1* results in reduced expression of the genes on the X-chromosome, while loss of *roX2* leads to MSL-independent upregulation of genes with male-biased testis-specific transcription. In *roX1 roX2* mutant, gene expression is strongly reduced in a manner that is not related to proximity to high-affinity sites.

## Results

### Expression of *roX1* and *roX2* is differentially regulated throughout the cell cycle

**Fig 1:**
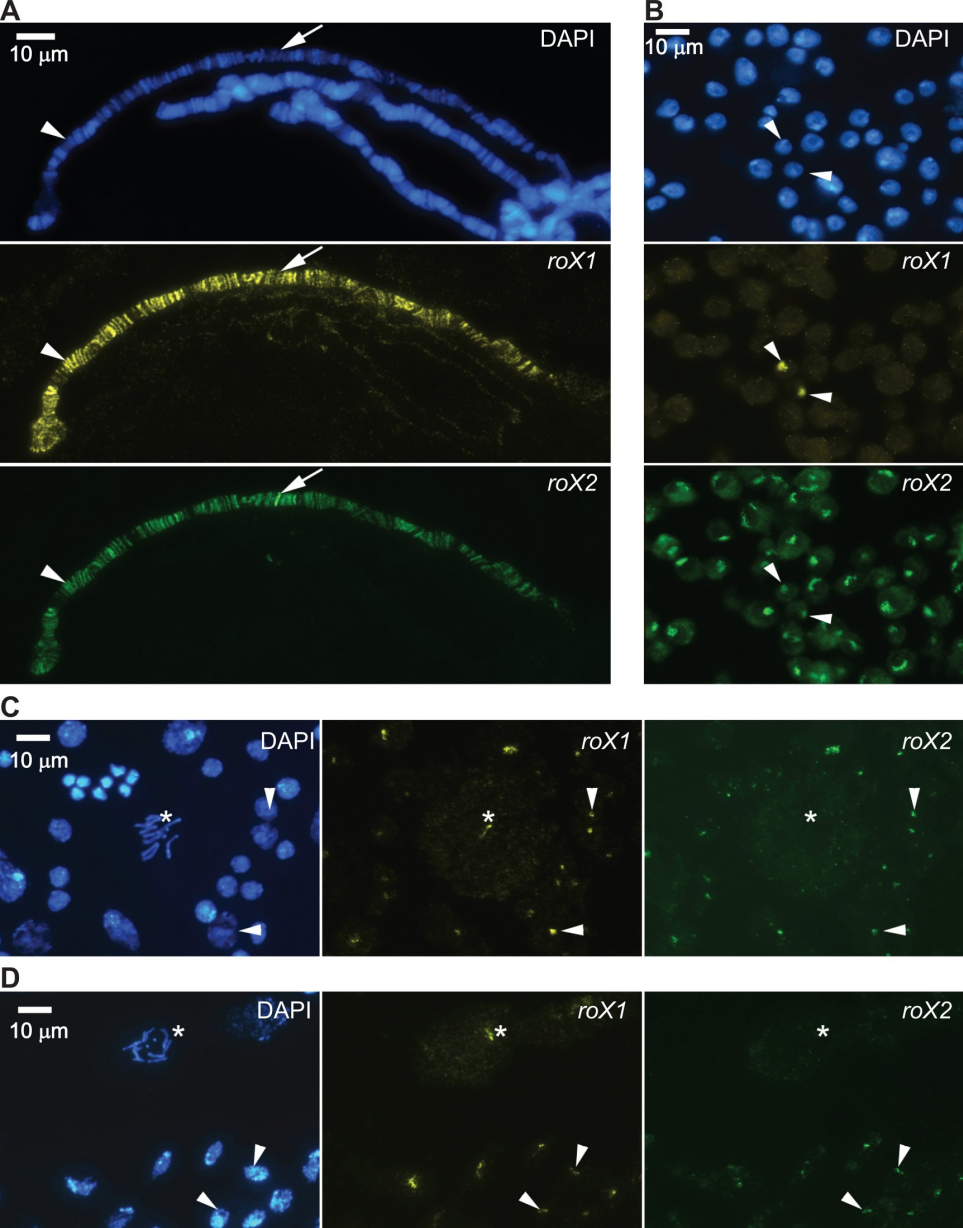
Targeting of *roX1* and *roX2* RNAs in indicated cell types. Results of RNA *in situ* hybridization with antisense probes against *roX1* (yellow) and *roX2* (green), with DAPI staining of DNA (blue). **(A)** *roX1* and *roX2* RNA target and colocalize on the X-chromosome in male 3^rd^ instar larvae salivary glands. The genomic loci of *roX1* (arrowhead) and *roX2* (arrow) are indicated. **(B)** *roX2* is the dominating *roX* species in S2 cells. The few cells with localized *roX1* domains (arrowheads) also show *roX2* targeting. **(C, D)** On metaphase chromosomes *roX1* but not *roX2* targets the distal part of the X-chromosome, in male 3^rd^ instar larvae neuroblasts **(C)** and in male 6 hour embryos **(D)**. Examples of interphase nuclei with colocalized *roX1* and *roX2* are indicated with arrowheads and the metaphase X-chromosome decorated by *roX1* is indicated by asterisks. More than 12 brain preparations and >9 embryos were examined. *roX1* was detected on the distal part of the X-chromosome in >70% of metaphase nuclei in both cases.

Initial evidence on localization of *roX* RNAs originates from immunostaining experiments on polytene chromosomes. Indeed, both *roX1* and *roX2* are expressed in salivary gland cells and co-localize on polytene chromosomes close to perfectly (Fig 1A). Overall, the intensities of *roX1* and *roX2* RNA *in situ* hybridization signals correlate closely, and the localization patterns along the X-chromosome are nearly identical, except at cytological band 10C, where the *roX2* signal is notably stronger than the *roX1* signal. As cytological band 10C is the location of the *roX2* gene, this implies that *roX2* is favored in MSL complexes targeting the *roX2* region rather than *roX1*. At the onset of dosage compensation in the early male embryo, expression of *roX* is differentially regulated [27, 28]. A burst of *roX1* transcription in the blastoderm stage is the initial step preceding assembly of the MSL complex. This occurs independently of *roX2* expression, which does not begin until 2 h after the MSL complex is first detectable on the X-chromosome. In Schneider 2 cells, *roX2* is expressed more strongly than *roX1* and is detectable by FISH in 95% of them, while *roX1* signals, although bright, are visible only in a small fraction of the cells [37]. We therefore asked whether *roX1* and *roX2* are expressed in different Schneider 2 cells. Simultaneous detection of both *roX* RNAs showed that the rare cells that express *roX1* also express *roX2* (Fig 1B). Therefore, in contrast to salivary glands and embryos, only a small fraction of S2 cells express both *roX* RNAs and all those expressing *roX2* also express *roX1*.

To investigate *roX* localization and targeting in cells undergoing mitosis we subjected neuroblasts of male larvae and 5–6 h embryos to RNA *in situ* hybridization analysis. While both *roX1* and *roX2* were clearly visualized in the “X-territory” in most interphase cells, only *roX1* signals were detected on the distal part of the metaphase X-chromosome (Fig 1C and 1D and S1A Fig). We also observed targeting of MLE to the distal part of the mitotic chromosome (S1A Fig), and such targeting by MSL2 and MSL3 has been previously shown [38, 39]. We conclude that expression and/or targeting of *roX* RNAs is differentially regulated depending on the cell type and cell cycle stage, and *roX1* RNA is the dominant *roX* RNA bound to the X-chromosome as part of MSL complexes during mitosis.

### Generation of new *roX2* mutant alleles

**Fig 2:**
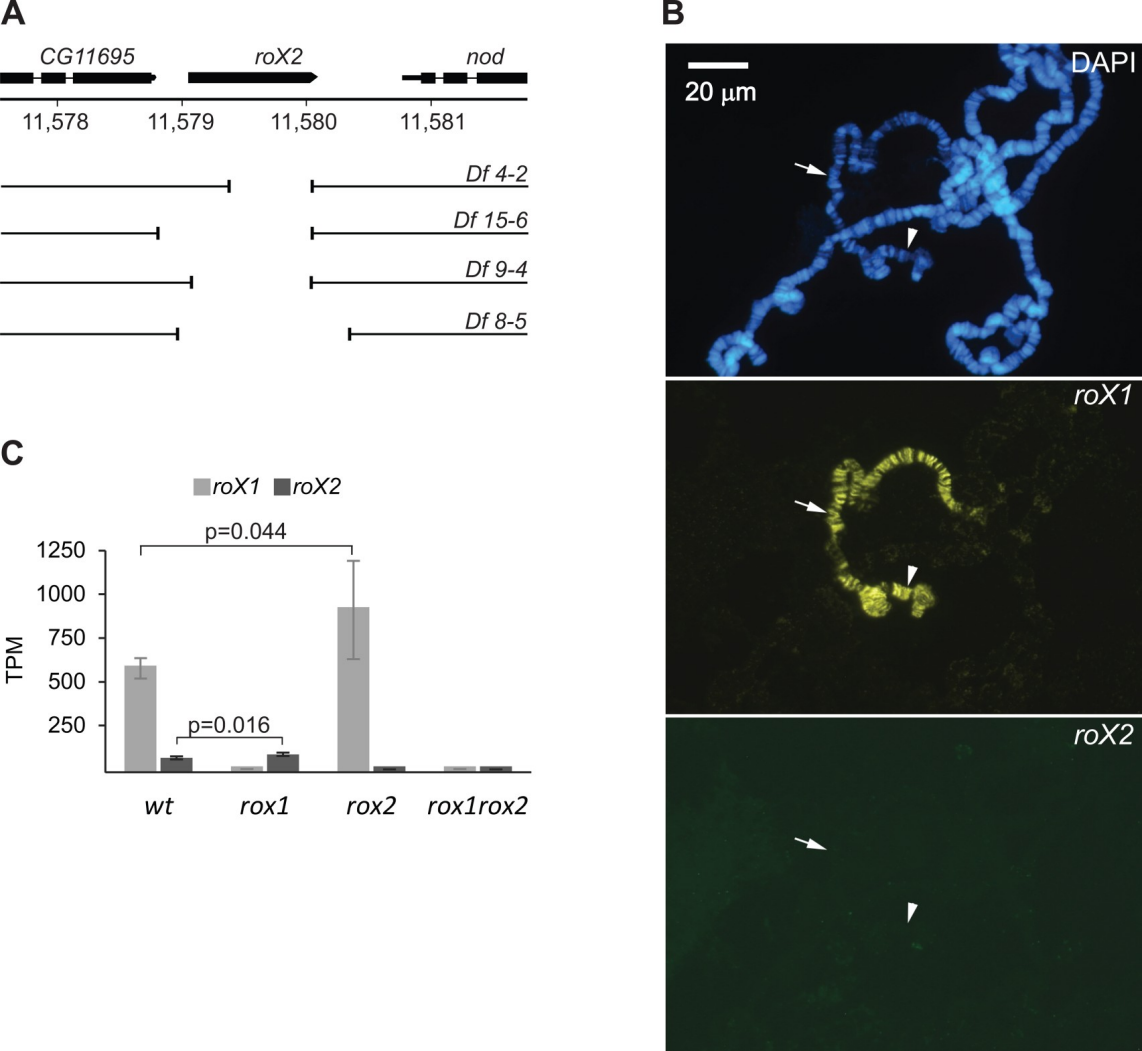
Novel deletion mutants of the *roX2* gene. **(A)** Map of *roX2* genomic locus and the extent of the novel CRISPR/Cas9 generated deletions. **(B)** Results of RNA *in situ* hybridization with antisense probes against *roX1* (yellow) and *roX2* (green) in salivary gland cells of the *roX2*^*9-4*^ strain. The genomic loci of *roX1* (arrowhead) and *roX2* (arrow) are indicated. **(C)** Average expression of *roX1* and *roX2* RNAs analyzed by high-throughput transcriptome sequencing. The X-axis shows transcripts per kilobase per million. Error bars indicate 95% confidence intervals. The number of biological replicates are n = 3 (wildtype), 3 (*roX1*), 4 (*roX2*) and 3 (*roX1 roX2*). Statistical significance was determined by Student’s t-test.

The *roX2* mutant allele *Df(1)52*, the most commonly used *roX2* loss-of-function allele, carries a deletion spanning a gene-dense region, including *roX2* [30]. Removal of this region is lethal, so it is compensated with a rescuing cosmid, frequently *P{w*^*+*^ *4Δ4*.*3}*. Nevertheless, *roX2* is not the only gene affected by the widely used combination *Df(1)52 P{w*^*+*^ *4Δ4*.*3}*, and genes carrying it differ considerably in genetic background from *roX1* and wild type flies. In a previous microarray analysis, potential background problems were solved by comparing *roX1 roX2* mutant flies with *roX2* flies as controls [40]. Here, to analyze differences in expression profiles of single (*roX1* and *roX2*) mutants and double (*roX1 roX2*) *roX* mutants we decided to create a deletion mutant of *roX2* without affecting adjacent genes. Such a mutant would permit analysis of single and double mutants using a *roX1*^*+*^ *roX2*^*+*^ strain as a control and facilitate various other genetic analyses. To create the desired mutant allele, we used the CRISPR-Cas9 technique to induce two double-strand breaks simultaneously in the *roX2* locus and recovered four *roX2* deletion mutant strains (Fig 2A and S2 Fig). All deletions in these mutants span the longest exon of *roX2*, including two conserved roX-boxes. As expected, all four mutant strains were viable and fertile. Further analysis was performed with the *roX2*^*9-4*^ allele, hereafter designated as the *roX2* mutant. This deletion does not uncover the intergenic regions flanking *roX2* and therefore it is less likely to affect the flanking genes *nod* and *CG11650*. The breakpoints are located almost precisely at the sites of double-strand breaks, deleting the region from 7 bp upstream of the annotated transcription start site to 60 bp upstream of the annotated gene end. RNA *in situ* hybridization confirmed the absence of *roX2* RNA in salivary glands (Fig 2B), while the *roX1* signal intensity and binding pattern were apparently unchanged in the *roX2* mutant. In larval brain of *roX1* mutants the *roX2* RNA was still observed in the X-territory of interphase cells, however it was not detected on the metaphase X-chromosome (S1B Fig). We recombined the newly made *roX2*^*9-4*^ allele with the *roX1*^*ex6*^ mutant allele [30] to obtain the *roX1*^*ex6*^ *roX2*^*9-4*^ double mutant flies, hereafter *roX1 roX2* mutant. As observed with other mutant alleles, removal of both *roX* RNAs resulted in high male-specific lethality beginning at the third instar larvae stage and continuing through pupal development, although a small number of adult males hatched.

### Chromosome-specific effects in *roX* mutants

The next experiments were designed to investigate the specific roles (if any) of the *roX* RNA species in dosage compensation and assess potential additional functions in regulation of gene expression. For this, we sequenced (using an Illumina platform) polyadenylated RNA from wildtype, *roX1* mutant, *roX2* mutant and *roX1 roX2* mutant 1^st^ instar male larvae. This developmental stage was chosen to minimize indirect effects of dosage compensation failure in the *roX1 roX2* mutant, as *roX1*^*ex6*^ *roX2*^*9-4*^ 1^st^ instar larvae are healthier than those of later stages. The four genotypes compared are not isogenic, however, the outcrosses as described in Material and methods ensure that the entire autosomal complement is heterozygous in all genotypes and half of it will have identical origin. Still, we cannot fully exclude that remaining differences in genetic background could be a contributing factor to the observed changes in expression for some genes.

**Fig 3:**
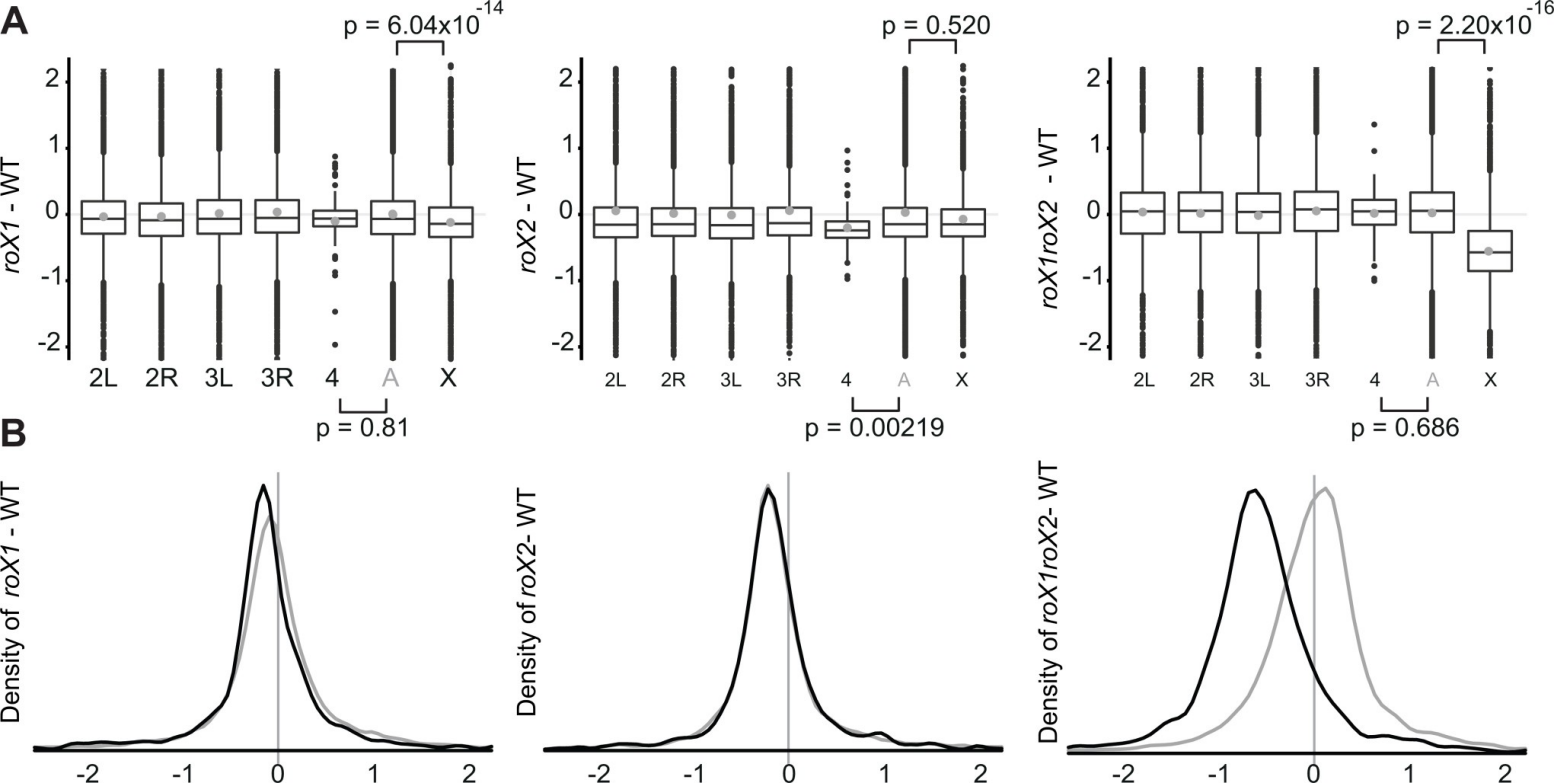
*roX* RNAs are required for proper transcription of genes on the 4^th^ and X-chromosomes. **(A)** Boxplots representing distributions of expression ratios for individual chromosome arms and chromosomes 2 and 3 combined (light grey A). The grey dots represent means of the samples. **(B)** Density plots of expression ratios for the X chromosome (black) and chromosomes 2 and 3 combined (grey). The vertical bar indicates the 0.

In wildtype larvae, *roX1* RNA was approximately ten times more abundant than in *roX2* mutant larvae (Fig 2C). Notably, we observed increases in abundance of both *roX* RNAs in response to absence of the other, but not establishment of wildtype *roX* levels, in the single mutants. More specifically, we recorded 89% reductions in *roX* RNA levels in the *roX1* mutant, while removal of *roX2* RNA (which normally constitutes only 7% of the total *roX* RNA complement) resulted in a 45% increase in *roX1* RNA abundance on average. Therefore, the single mutants differ considerably in levels of *roX* RNA. Moreover, although viability and fitness are not affected in either of the single mutants, the efficiency of dosage compensation is significantly compromised in the *roX1* mutant. The average log2 expression ratio of the X-chromosome in this mutant was -0.13, corresponding to an 8.6% reduction in average expression of X-chromosome genes relative to genes on the four major autosomes. In the *roX2* mutant, the average expression ratio for X-chromosome genes was lower than that of autosomal genes, but density distributions for X and autosomal expression ratios were very similar (Fig 3A and 3B and S3 Fig). A Mann-Whitney U-test confirmed that the two populations cannot be differentiated in terms of these expression parameters, so global X-chromosome transcription is not significantly affected in the *roX2* mutant. In conclusion, the *roX2* mutant shows no lack of compensation and has *roX* levels comparable or even higher than wildtype. Thus, it is not clear whether the total amount of *roX* or the type of *roX* is responsible for the observed reduction in average expression of X-chromosome genes in the *roX1* mutant. The results also implies that the observed increase in levels of *roX1* RNA in the *roX2* mutant (Fig 2C) does not lead to hyper-activation of the X-chromosome but is enough to maintain proper X-chromosome expression.

We and others have previously shown that in absence of *roX* RNAs, the MSL-complex become less abundant on the X-chromosome and relocated to heterochromatic regions including the 4^th^ chromosome [14, 30, 37, 40]. In fact, the fourth chromosome is related to the X-chromosome and evolutionary studies have shown that the 4^th^ chromosome was ancestrally an X-chromosome that reverted to an autosome [41, 42]. Importantly, upon analysis of the 4^th^ chromosome we detected weak but significant downregulation of genes on the fourth chromosome as a specific consequence of *roX2* deletion (Fig 3A), but not the previously reported downregulation of the fourth chromosome in the *roX1 roX2* mutant flies [43].

As expected, strong downregulation of X-linked genes occurred in the *roX1 roX2* mutant (Fig 3C). However, it was more severe (a 33% reduction relative to wildtype levels) than previously reported in microarray studies [40], and following RNAi depletion of MSL proteins [9, 43–45]. The distribution plot shows that the vast majority of genes were downregulated in the *roX1 roX2* mutant and the entire distribution of X-chromosomal gene expression was shifted approximately -0.56 on log2 scale relative to the expression of genes on the four major autosomal arms.

### Dosage compensation of genes in *roX* mutants depends on their location

**Fig 4:**
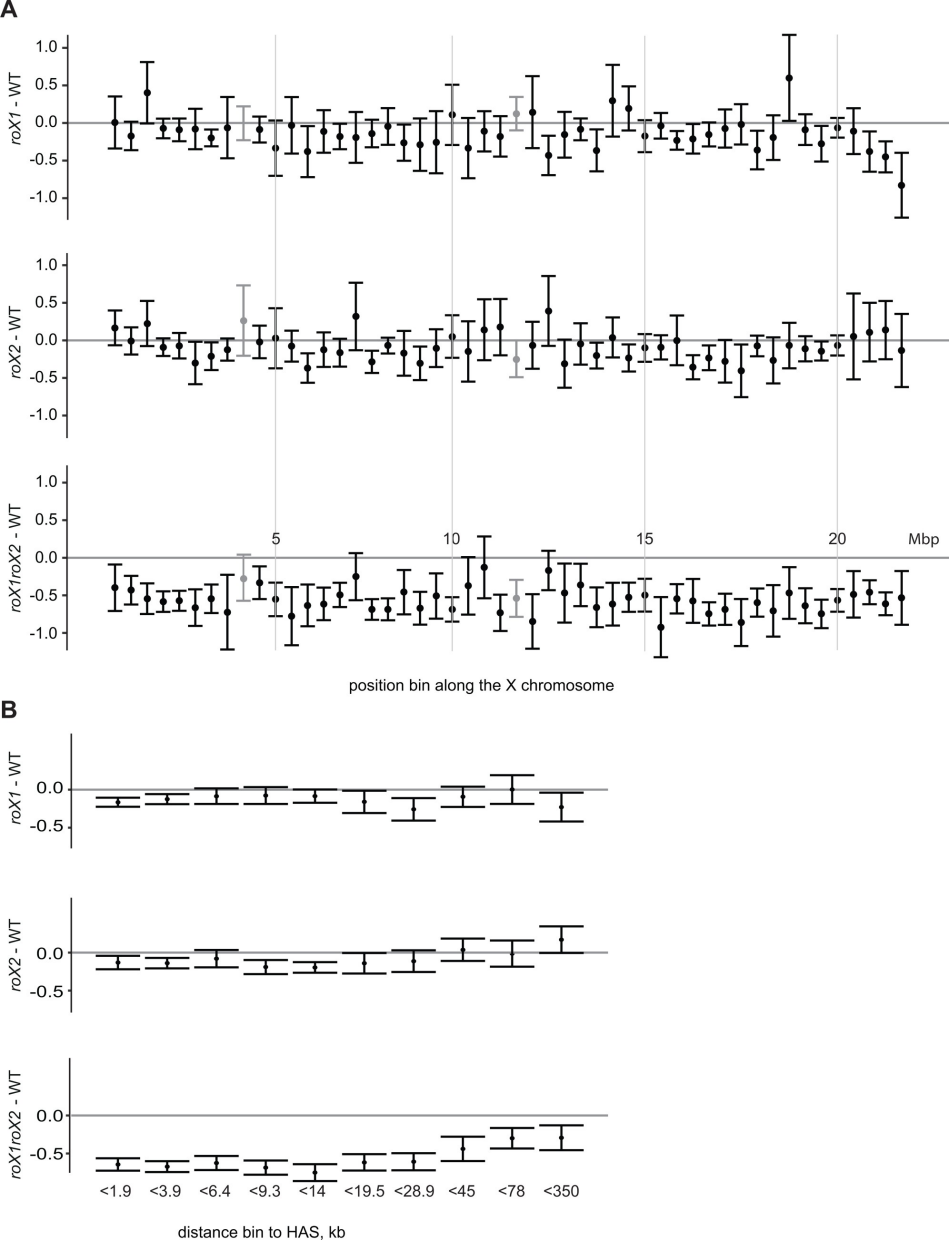
Genes far from high-affinity sites require both *roX* RNAs for proper expression. **(A)** Expression ratio distribution along the X-chromosome, divided into 50 bins of equal length. Genes were assigned to bins according to coordinates of their centers. The grey bars indicate bins containing *roX1* and *roX2* genes. **(B)** Expression ratios of all X chromosomal genes plotted against distance to high-affinity sites split into bins with equal number of genes. The error bars represent 95% confidence intervals.

The expression ratios of X-linked genes varied widely, especially in the *roX1 roX2* mutant (Fig 3C). It has been proposed that MSL complexes are assembled at the sites of *roX* RNA transcription, then spread to the neighboring chromatin in *cis* direction, as well as diffusely, gradually binding to more distant loci. In addition, our *in situ* hybridization results indicate enrichment of *roX2* RNA at cytological region 10C. We therefore tested if dosage compensation has a distinct spatial pattern along the X-chromosome. We observed some clustering of genes related to sensitivity to *roX1* or *roX2* RNAs, but it appeared to be randomly distributed spatially, except for a gradual decrease in expression of genes in the proximal X-chromosome region in the *roX1* mutant, and the 10C region in the *roX2* mutant (Fig 4A).

A number of studies have estimated that the MSL complex binds specifically to roughly 250 chromatin entry sites, high-affinity sites (HAS) or Pion-X sites. Since *roX* RNAs are important for the spreading of the MSL complex from these high-affinity sites we asked whether the extent of genes’ differential expression in *roX* mutants correlates with their distances from these sites. Dot plots of genes’ expression ratios against their distances from HAS or Pion-X sites showed weak trends, but were difficult to interpret due to high variation (S4 Fig). Thus, for more informative visualization we grouped the genes into bins with increasing distance from HAS (Fig 4B). In *roX1* mutant, the average expression ratio was not significantly affected by the distance from HAS. This was also true for genes located within approximately 30 kb from HAS in *roX2* and *roX1 roX2* mutant. However, more remote genes had higher average expression ratios in *roX2* and *roX1 roX2* mutant, and thus are less suppressed in the double mutant and even upregulated in the *roX2* mutant. On polytene chromosomes in the *roX1 roX2* mutant we still observed MSL targeting on the X-chromosome, but only at HAS [14]. This might suggest that genes close to HAS would retain dosage compensation function also in the absence of *roX* RNAs. On the contrary, our results show that genes within approximately 30 kb from HAS are strongly and equally affected while genes more distal to HAS are less sensitive to the absence of *roX* and absence of bound MSL complex.

### The *roX* sensitivity of genes depends on the MSL complex binding strength

**Fig 5:**
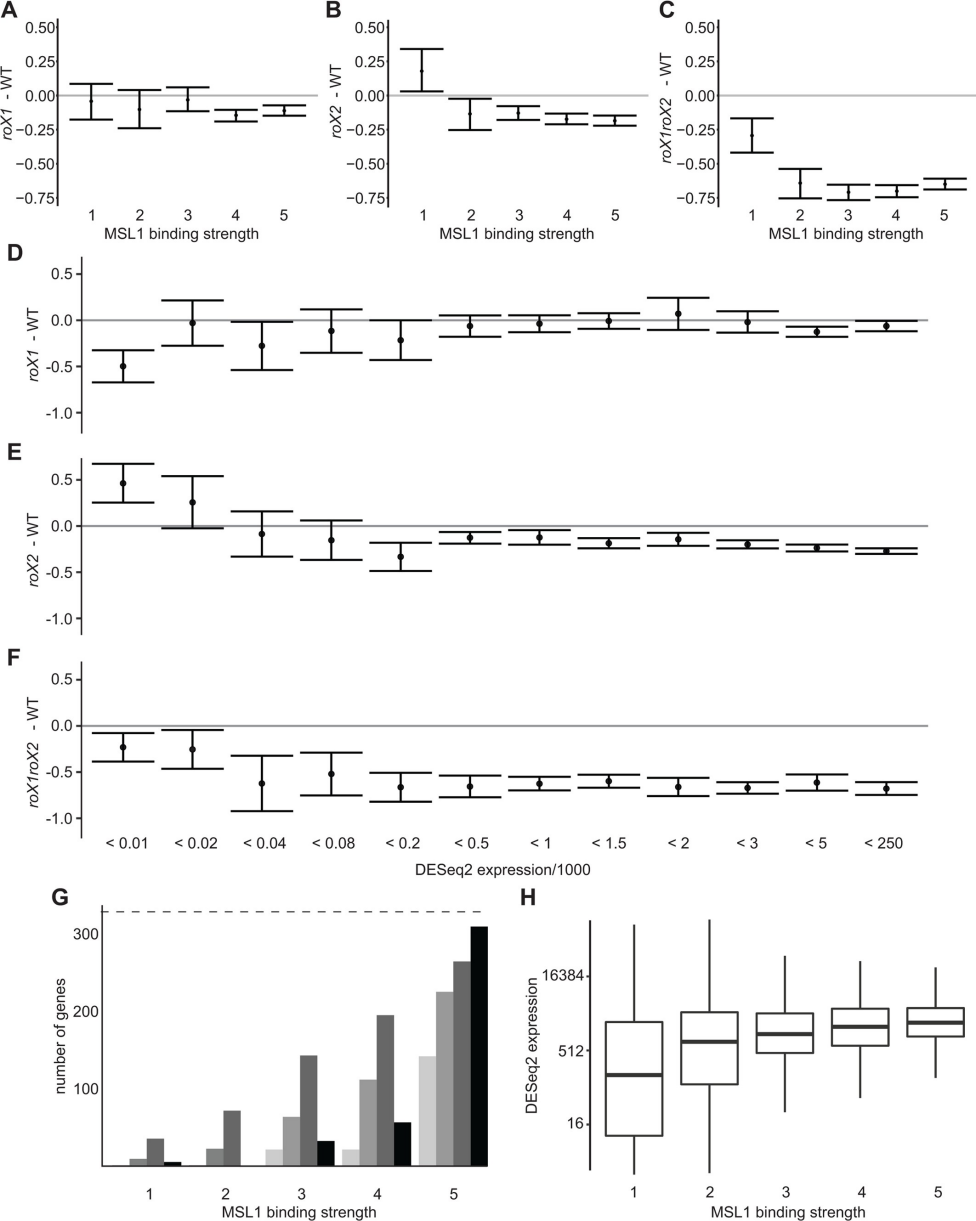
Highly-expressed genes require high MSL complex levels for proper expression. **(A-C)** Average expression ratios of X chromosomal genes grouped in equal sized bins based on their MSL1 binding strength (1 lowest to 5 highest); **(A)** *roX1* –WT, **(B)** *roX2* –WT, **(C)** *rox1 roX2*—WT. **(D-F)** Average expression ratios plotted against binned expression values. The error bars indicate 95% confidence intervals; **(D)** *roX1* –WT, **(E)** *roX2* –WT, **(F)** *roX1 roX2*—WT. **(G)** Numbers of genes located within 2, 5 and 10 kb of HAS (three grey bars) and genes overlapping with MSL1 peaks in the *roX1 roX2* mutant (black bar), plotted for wild type MSL1-binding bins. The dashed line at the top represents the total number of genes in each MSL binding bin (n = 328). **(H)** Boxplots showing the distribution of expression in the five MSL1 binding bins.

We next asked if the *roX*-dependent dosage compensation depends on the binding strength of the MSL complex, using publicly available chromatin immunoprecipitation data on MSL1, MOF and MSL3 [46] to correlate with our differential expression data (Fig 5A–5C and S5 Fig). All X-chromosome genes were ranked in order of increasing MSL complex enrichment and divided into five bins with equal numbers of genes. Thus, bin 1 included unbound and weakly bound genes, while bin 5 included genes highly enriched in MSL proteins. We found that genes in bins 1 and 2 responded more variably to removal of either or both *roX* RNAs, a pattern that is probably related to their low expression levels (Fig 5H). In the single *roX* mutants, expression ratios did not correlate with enrichment of MSL proteins (Fig 5A and 5B and S5A, S5B, S5D and S5E Fig), indicating that MSL complex-regulated genes uniformly respond to the absence of one *roX* RNA, regardless of the enrichment levels in wildtype flies. Strikingly, strong and significant upregulation of genes classified as non- or weakly MSL complex-binding was detected in the *roX2* mutant, similarly to genes located far from HAS (Fig 5B and S5B and S5E Fig). In *roX1 roX2* mutant, these weakly MSL complex-binding genes are still suppressed, but much less than strongly binding genes. Since the MSL complex is still enriched at HAS in the absence of *roX* it is surprising that dosage compensation by *roX* RNA-free MSL complexes has low efficiency even for genes with the highest MSL enrichment. The genes highly enriched in MSL1 and MSL3 (bin 5) were slightly less down-regulated, but this trend was not seen with MOF enrichment bins (S5F Fig).

Since genes with low MSL complex-binding levels are less suppressed than others in the *roX1 roX2* mutant, and upregulated in the *roX2* mutant, we asked whether dosage compensation in the absence of *roX* depends on genes’ expression level. For this, we divided the X-chromosome genes into 12 equally sized bins according to their expression levels. In accordance with observations regarding genes that weakly bind the MSL complex, we observed upregulation of weakly expressed genes in the *roX2* mutant and less pronounced reduction in their expression in the *roX1 roX2* mutant (Fig 5D–5F).

High-affinity sites are defined as those that retain incomplete MSL complexes in *msl3*, *mle* or *mof* mutants [45, 47–51], and it has been suggested that MSL complex-binding is directed by hierarchical affinities of target sites [49, 50]. In the *roX1 roX2* mutant we observed more pronounced reductions in MSL complex abundance on the male X-chromosome than those reported in *msl3*, *mle* or *mof* mutants, but the remaining MSL targets in the *roX1 roX2* mutant were highly reminiscent of those described in *msl3*, *mle* and *mof* mutants [14, 30]. We observed reduced expression of strongly MSL-binding genes in the *roX1 roX2* mutant, which is intriguing as these genes are assumed to retain the MSL complex [14]. Thus, to test the suggestion, we explored correlations between the MSL binding bins and 263 high affinity sites defined by targeting in *mle*, *mof* or *msl3* mutants, or following their depletion [45, 51, 52]. In parallel we analyzed the 208 peaks we previously identified in the absence of *roX1 roX2* [14]. The previously defined 208 peaks in the *roX1 roX2* mutant overlap 405 genes on the X-chromosome, 309 of which are among the 328 genes in bin 5 (Fig 5G). We conclude that the 208 MSL peaks defined in the *roX1 roX2* mutant correspond more strongly with genes in the highest MSL binding class than the previously defined HAS do (Fig 5G). Intriguingly, expression of X chromosomal genes also correlates with MSL1 binding enrichment (Fig 5H), and thus overlap with HAS. This suggests that the distribution of MRE motifs and consequently MSL complex-binding is governed by gene expression in a manner that promotes adequate dosage compensation in males.

### *roX* sensitivity and replication timing

**Fig 6:**
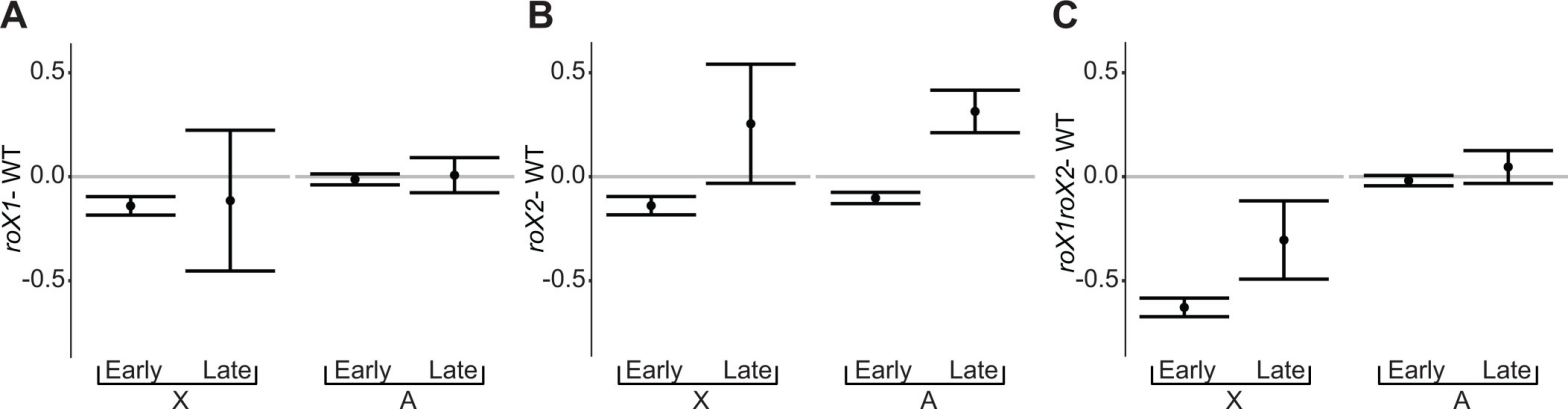
Differential effects of *roX* mutations on early and late replicating genes. Average expression ratios of X chromosomal (X) and autosomal (A) genes in; **(A)** *roX1* –WT, **(B)** *roX2* –WT, **(C)** *roX1 roX2*—WT. The genes are grouped by their replication time in S2 cultured cells and the expression ratios are calculated from the RNA-seq analysis on first instar larvae. The error bars represent 95% confidence intervals.

In higher eukaryotes replication timing is connected to the chromatin landscape and transcriptional control [53]. Generally, early replicating regions are associated with active transcription [54–56] whereas late replicating regions are associated with inactive regions and heterochromatin [57]. Genome-wide studies on cultured *Drosophila* cells have revealed dependency of male-specific early replication of the X-chromosome on the MSL complex [56, 58]. We therefore asked whether X-chromosomal or genome-wide sensitivity to a specific *roX* mutant condition correlate with replication timing. Using available data on replication timing from analyses of S2 and DmBG3 (male) and Kc167 (female) cells [58] we classified the genes as early or late replicating. Based on our RNA-seq data we then calculated expression ratios for genes grouped by their chromosome location (autosomal or X-chromosomal) and their replication timing as determined in the three cell types. Conceivably, early and late X chromosomal replication domains (determined from analyses of S2 and DmBG3 male cell cultures) are respectively associated with genes bound and unbound by the MSL complex, and thus are affected in similar manners by *roX* mutations (Fig 6 and S6 Fig). In female Kc167 cells the relation between sensitivity to *roX* and replication timing is generally similar to that observed in male cell cultures. However, in Kc167 cells the X-chromosome has a slightly different pattern of replication domains, which shifts the average expression ratio (Fig 6 and S6 Fig). In particular, the distribution of distinctively upregulated X chromosomal genes in the *roX2* mutant only corresponds with the distribution of late-replication regions in male cells. Notably, in larval neuroblasts and embryonic cells (Fig 1C and 1D), we only detected *roX1* RNA (no *roX2* RNA) on mitotic X-chromosomes, suggesting that *roX1*-containing MSL complexes mediate dosage compensation in the G1 phase, when replication timing is established [59]. It is tempting to speculate that selective transmission of *roX1*-containing MSL complexes through mitosis enables the cells to quickly and efficiently establish the correct chromatin state and hence maintain correct replication timing.

### Testis-biased genes are derepressed in *roX2* mutant

Transcription upregulation of the X-chromosome in the *roX2* mutant is associated with genes classified as having low expression levels, late replication and weak MSL complex-binding. We asked if this observed upregulation is caused by mis-targeting of MSL complexes associated with excess of *roX1*, *i*.*e*., if the upregulated genes are enriched in MSL complexes due to increases in *roX1* levels and/or loss of *roX2*. To test this possibility, we assessed relative enrichments of MSL1 and H4K16ac on the upregulated genes by ChIP-qPCR analyses. In the *roX2* mutant, none of the eight genes we tested became targeted by MSL1 or enriched in H4K16ac at a comparable level to known MSL target genes (S7 Fig). In contrast, enrichment levels were similar to those detected on the autosomal control genes *RpS3* and *RpL32*. We therefore conclude that stimulation of weakly expressed X chromosomal genes in the *roX2* mutant is not mediated by induced targeting of the MSL complex.

**Fig 7:**
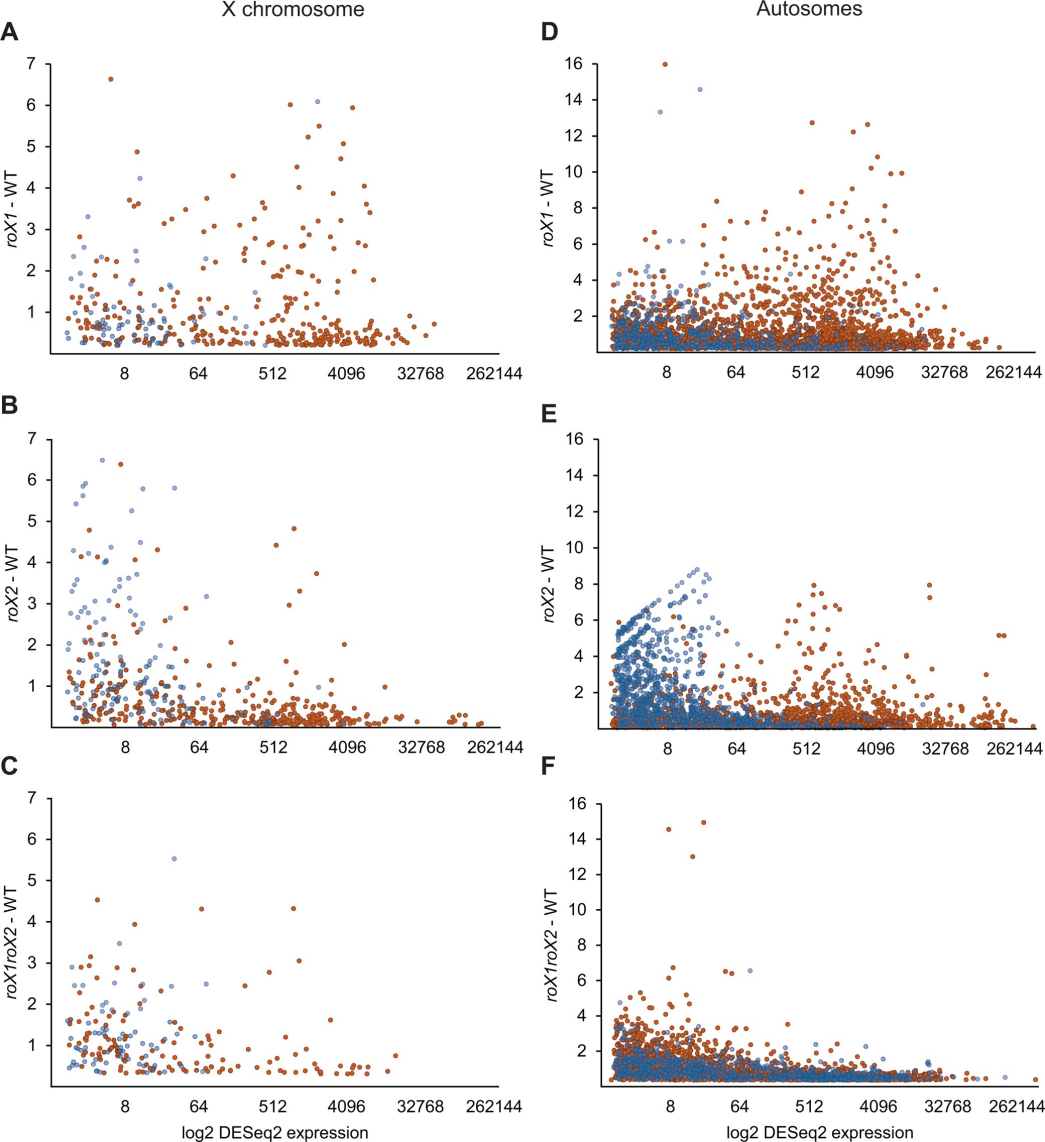
Testis-biased genes are upregulated in the *roX2* mutant. Expression ratios plotted against log2-converted expression value for testis-biased genes (blue) and all other genes (orange). **(A-C)** show X chromosomal genes; **(A)** *roX1* –WT, **(B)** *roX2* –WT, **(C)** *roX1 roX2*—WT. **(D-E)** show autosomal genes; **(D)** *roX1* –WT, **(E)** *roX2* –WT, **(F)** *roX1 roX2*—WT. Testis-biased genes were determined as genes with testis enrichment scores exceeding 4 according to the FlyAtlas2 expression database.

Further analysis of upregulated genes in the *roX2* mutant showed that they included not only X chromosomal genes but also late-replicating autosomal genes. This, together with the absence of MSL complex-enrichment on these genes, indicates that the upregulation is a *roX2*-specific effect and at least partly separable from MSL complex-mediated gene regulation. Intriguingly, we discovered that these upregulated genes in the *roX2* mutant strain include high proportions of genes (both X-chromosomal and autosomal) with male-biased testis-specific transcription (Fig 7). Whether *roX2* has a specific role in transcriptional regulation of genes involved in spermatogenesis or the observed phenomenon is an indirect consequence of *roX2* mutation is an intriguing question that warrants further investigation.

## Discussion

The dosage compensation machinery involving *roX1* and *roX2* RNAs provides a valuable model system for studying the evolution of lncRNA-genome interactions, chromosome-specific targeting and gene redundancy. LncRNAs differ from protein coding genes and are often less conserved at the level of primary sequence, as expected due to their lack of protein-coding restrictions. Like those encoding other lncRNAs, rapid evolution, *i*.*e*., low conservation of the primary sequences of *roX* genes has complicated comparative studies [24, 60]. Despite their differences in length and primary sequences, *roX1* and *roX2* have also been considered functionally redundant in *Drosophila melanogaster*. However, remarkably considering their rapid evolution and apparent redundancy, orthologs for both *roX1* and *roX2* have been found in all of 26 species within the *Drosophila* genus with available whole genome assemblies [60]. Models that explain evolutionarily stable redundancy have been proposed [61] suggesting that the presence of both *roX1* and *roX2* in these diverged species may be attributable to differences in targets, affinities and/or efficiency or additional functions.

On polytene chromosomes, binding patterns of *roX1* and *roX2* are more or less indistinguishable, except in region 10C where *roX2* is almost exclusively present. In the *roX2* mutant, genes located in the 10C bin are on average downregulated, but similar downregulation of genes in many other bins is observed, so the effect cannot be directly attributed to loss of *roX2*. In wildtype 1^st^ instar larvae, levels of *roX1* RNA are much higher than levels of *roX2* RNA. Interestingly, in *roX1* mutant larvae the absolute amount of *roX2* RNA increases, but only to ~10% of wildtype levels of total *roX* RNA. This appears sufficient to avoid lethality, but still causes a significant decrease in X-chromosome expression. However, despite the huge difference in amounts, not only in number but even more considering the size of the two *roX* RNAs, the staining intensities of *roX* RNA on *roX1* mutant and wildtype polytene chromosomes seem to be roughly equal. On mitotic chromosomes we only observed *roX1* RNA in the MSL complexes bound to the distal X-chromosome and this binding is not redundant. This indicates that just after cell division *roX1* RNA will be the dominating variant in assembled MSL complexes. Taken together, our results suggest that *roX2* RNA has higher affinity than *roX1* RNA for inclusion in MSL complexes. Moreover, varying amounts of the two species with different affinities at given cell cycle stages may support proper transmission, spreading of assembled MSL complexes and maintenance of appropriate levels of the complexes.

It should be noted that some male *roX1 roX2* mutant escaped, so loss of *roX* is not completely male-lethal, unlike loss of *mle*, *msl1*, *msl2*, *msl3* or *mof* [29–31, 62]. The complete male lethality in these mutants is attributed to reductions in dosage compensation that have been measured in several studies and observed not only in *msl* mutants but also following RNAi-mediated depletion of MSL proteins [9, 43–45]. Notably, the average reduction of X-chromosome expression, relative to wildtype levels, calculated in these cases has varied from ca. 20 to 30%; substantially less than the 35% reduction we observed in the *roX1 roX2* mutant. Some of the reported differences may be due to use of different techniques and bioinformatics procedures (including use of different cut-offs for expression and developmental stages). However, the reasons why some males can survive the very dramatic imbalance observed in expression of a large portion of the genome are unclear. Furthermore, the reduction in expression of X-chromosome genes observed in the *roX1* mutant is not accompanied by any reported phenotypic changes, indicating that *D*. *melanogaster* has high intrinsic ability to cope with significant imbalances in X-chromosome expression. We speculate that in parallel with a compensation mechanism that addresses dosage imbalances the fly has evolved a high degree of tolerance to mis-expression of the X-chromosome.

The 4^th^ chromosome in *D*. *melanogaster* (the Muller F-element) is related to the X-chromosome. Evolutionary studies have shown that sex chromosomes do not always represent terminal stages in evolution—in fact, the 4^th^ chromosome was ancestrally an X-chromosome that reverted to an autosome [41, 42]. Moreover, the fly shows high and unusual tolerance to dosage differences [63] and mis-expression [8, 64–66] of the 4^th^ chromosome (although much smaller than the tolerance to those of the X-chromosome). These observations suggest that tolerance of mis-expression is a common outcome in the evolution of sex-chromosomes and this property has been retained with respect to the 4^th^ chromosome, even after its reversion to an autosome. We propose that high tolerance of mis-expression in the absence of full functional dosage compensation may be selected for during evolution of sex-chromosomes. This is because gradual degeneration of the proto-Y chromosome will be accompanied by an increasing requirement to equalize gene expression between a single X- (in males) and two X-chromosomes (in females), but changes in genomic location of highly sensitive genes will be favored during periods of incomplete (or shifting) dosage compensation. On transcript level, responses to reductions in dosages of X-chromosome genes have been found to be similar to those of autosomal genes [67]. Thus, potential mechanisms for the higher tolerance are post-transcriptional compensatory mechanisms or selective alterations in gene composition (changes in genomic locations), similar to those proposed for the observed demasculinization of the *Drosophila* X-chromosome [68].

Prompted by the strong relationship between orchestration of the X- and 4^th^ chromosomes by the MSL complex and POF system [2, 14, 69–71], respectively, we also measured effects of *roX* suppression on chromosome 4 expression in *roX* mutants. We observed weak but significant reduction of expression in the *roX2* mutant, but the cause of this reduction remains elusive. In *roX2* mutant we also observed transcriptional upregulation of X-chromosome genes classified as having low expression levels, late replication and weak MSL complex-binding. The loss of *roX2* resulting in MSL complexes only including *roX1* RNA might alter the spreading properties. We therefore hypothesized that the observed upregulation might be caused by mis-targeting of the MSL complex in the absence of *roX2*. However, our ChIP experiment revealed no enrichment of MSL complexes on these genes, and our results rather suggest that *roX2* directly or indirectly restricts expression of these male-biased genes independently of its role in the MSL complex.

It is well known that *roX* RNAs are important for spreading of the MSL complex in regions between HAS [11, 14]. It is therefore surprising that loss of *roX* causes a relatively even reduction in expression of X-chromosomal genes and the decrease is not more dramatic with larger distances, as would be expected for reductions in spreading capacity. Indeed, observed reductions in expression were smaller for genes located far from HAS than for closer genes. A possible explanation is that expression of these genes is compensated by an MSL-independent mechanism. It has been previously shown that most genes on the X-chromosome are dosage-compensated [9, 72, 73], but a subset are not bound by the MSL complex and do not respond to its depletion [74]. Our results corroborate these findings since loss of *roX* RNA in the *roX1 roX2* mutant had little effect on the expression of genes classified as having weak MSL complex binding, clearly indicating that at least one other mechanism is involved. The results further show that high-affinity sites, as defined by MSL-targets in the absence of *roX1* and *roX2*, are highly correlated to genes with the highest MSL binding levels. Therefore, sites targeted in the absence of *roX* provide a more stringent definition of HAS, with stronger correlation to genes bound by high levels of MSL complex, than targets in the absence of *mle*, *mof* or *msl3*.

The increase in expression mediated by the MSL complex is considered a feed-forward mode of regulation, and appears to be more or less equal (ca. 35%) for all MSL-bound genes [9]. Evidently, highly expressed genes need a stronger increase in transcription than weakly-expressed genes. Our results suggest that dosage compensation is a stochastic process that depends on HAS distribution and is correlated with expression levels. Evolutionary analysis has shown that newly formed X-chromosomes acquire HAS, putatively via rewiring of the MSL complex by transposable elements and fine-tuning of its regulatory potential [75, 76]. Such a dynamic process may be required for constant adaptation of the system. Highly expressed genes tend to accumulate HAS in their introns and 3´UTRs, and thus bind relatively high amounts of MSL complex, thereby stimulating the required increase in expression. This also implies that the gene organization on X-chromosomes is under more constraints than autosomes.

This study presents, to our knowledge, the first high-throughput sequencing data and analysis of transcriptomes of *roX1*, *roX2* and *roX1 roX2* mutant flies. The results reveal that *roX1* and *roX2* fulfill separable functions in dosage compensation in *D*. *melanogaster*. The two RNA species differ in both transcription level and cell-cycle regulation.

In third instar larvae, *roX1* is the more abundant variant and the variant that is included in MSL complexes transmitted physically associated with the X-chromosome in mitosis. Loss of *roX1*, but not loss of *roX2*, results in decreased expression of genes on the X-chromosome, albeit without apparent phenotypic consequences. Loss of both *roX* species leads to a dramatic reduction of X-chromosome expression, but not complete male lethality. Taken together, these findings suggest that high tolerance for mis-expression of X-chromosome genes has evolved. We speculate that it evolved in parallel with dosage compensation mechanisms and that it may be a common property of current and ancient sex-chromosomes.

The *roX* RNAs are important for spreading of the MSL-complex from HAS, but the reduction of X-chromosome expression in *roX1 roX2* mutant is not affected by the need for spreading, *i*.*e*., distance from HAS. In addition, the genes targeted by the MSL complex in the *roX1 roX2* mutant also show strongly reduced expression. Our results suggest that the function of the MSL complex which is still present at HAS is compromised in the *roX1 roX2* mutant and that the dosage of distant genes is compensated by an alternative, unknown, mechanism. We propose that dosage compensation is a stochastic process that depends on HAS distribution. Creation and fine-tuning of binding sites is a dynamic process that is required for constant adaptation of the system. Highly expressed genes will accumulate and be selected for strong HAS (and thus bind more MSL complex) since they require high levels of bound MSL complex for the required increases in expression.

## Material and methods

### Fly strains and *roX2* mutant generation

Flies were cultivated and crossed at 25°C in vials containing potato mash-yeast-agar. The *roX1*^*ex6*^ strain [77] was obtained from Victoria Meller (Wayne State University, Detroit). The new *roX2* mutant alleles were generated by CRISPR/Cas9 genome editing using a previously outlined strategy [78]. Briefly, we constructed a transgenic fly strain expressing two gRNAs in the germline, which are designed to induce double-strand breaks 7 bp upstream of the *roX2* transcription start site and 63 bp upstream of the annotated transcription termination. Males with the transgenic gRNA construct were crossed with *y*^*2*^ *cho*^*2*^ *v*^*1*^*; attP40{nos-Cas9}/CyO* females. The male progeny of this and subsequent two crosses were crossed individually to *C(1)DX*, *y*^*1*^ *w*^*1*^ *f*^*1*^ females. Strains with deletions spanning *roX2* were identified by PCR-based screening followed by sequencing, using primers and gRNA oligos listed in S1 Table. Males carrying a *roX2*^*9-4*^ deletion with the final genotype *y*^*1*^ *cho*^*2*^ *v*^*1*^ *roX2*^*9-4*^ were crossed with *y*^*1*^ *w*^*1118*^ *roX1*^*ex6*^ females to obtain recombinant *roX* double mutant X-chromosome *y*^*1*^ *w*^*1118*^ *roX1*^*ex6*^ *v*^*1*^ *roX2*^*9-4*^. This means that the crossover occurred between *cho* and *v* genes.

### RNA *in situ* hybridization

Previously described procedures were used in RNA-fluorescent in situ hybridization (FISH) analyses, and preparation of both salivary gland squashes [79] and larval brain squashes [80], following protocol 1.9, method 3, for the latter. Schneider’s line 2 cells were treated prior to hybridization as also previously described [37]. For embryo staining, *y*^*1*^ *w*^*1118*^ embryos were collected on apple juice-agar plates for 1 hour and incubated for 5–6 hours at 25°C. Squashes were prepared as follows: each embryo to be stained was manually dechorionated and transferred onto a cover slip. The vitelline membrane was pricked with a fine needle and a drop of 2% formaldehyde, 0.1% Triton X-100 in 1× PBS was added immediately. After 2 minutes, the solution was removed with a pipette and a drop of 50% acetic acid, 1% formaldehyde solution was added. After another 2 minutes incubation, a polylysine slide was placed over the cover slip. To spread the cells, the cover slip was gently pressed and then flash-frozen in liquid nitrogen. After removal of the coverslip the slide was immersed in 99% ethanol and stored at -20°C prior to hybridization. Antisense RNA probes for *roX1* (*GH10432*) and *roX2* (*GH18991*) were synthesized using SP6 RNA Polymerase (Roche) and DIG or Biotin RNA Labelling Mix (Roche), respectively. Primary antibodies were sheep anti-digoxigenin (0.4 mg/mL; Roche) and mouse anti-biotin (1:500, Jackson ImmunoResearch). The secondary antibodies were donkey anti-mouse labelled with Alexa-Fluor488 and donkey anti-sheep labelled with Alexa555 (Thermo Fisher Scientific).

### Preparation of RNA library, sequencing and data treatment

To obtain 1^st^ instar male larvae we collected 80–100 virgin females of the following genotypes: *y*^*1*^ *w*^*1118*^ (used as *wild type*), *y*^*1*^ *w*^*1118*^ *roX1*^*ex6*^ (*roX1* mutant), *y*^*1*^ *cho*^*2*^ *v*^*1*^ *roX2*^*9-*4^ (*roX2* mutant), and *y*^*1*^ *w*^*1118*^ *roX1*^*ex6*^ *v*^*1*^ *roX2*^*9-4*^*/FM7i*, *P[w*^*+mC*^ *ActGFP]JMR3* (*roX1 roX2* mutant). The females were crossed with 50–80 *FM7i*, *P[w*^*+mC*^ *ActGFP]JMR3/Y* males. Non-GFP 1^st^ instar larvae were collected, 20 per sample. The collected larvae were flash-frozen in liquid nitrogen and stored at -80°C. Total RNA was extracted with 1 mL of Tri Reagent (Ambion) per sample, and libraries were prepared with a TruSeq RNA Sample Prep Kit v2 (Illumina) according to the manufacturer’s instructions. In total, three wildtype, *roX2* mutant and *roX1 roX2* mutant biological replicates were prepared and four *roX1* mutant replicates. The samples were sequenced using a HiSeq2500 instrument at SciLife lab (Uppsala) and 125 bp long paired-end reads were obtained, and mapped to *Drosophila melanogaster* genome version 6.09 using STAR v2.5.1b with default settings. Read counts were obtained with HTseq version 0.6.1 using htseq count with default settings. The samples used for the analysis had 29.3–56.2 M reads with STAR mapping quality values of 22.9–52.1 and mean mapping coverage of 201–497. After removing genes with low read counts, means of the total expression of the four major autosome arms were centered to zero. Genes were annotated using the dmelanogaster_gene ensembl dataset from BioMart, *Dm* release 6.17.

### Differential expression analysis

Fold-differences in expression of genes among the investigated genotypes were calculated using the DESeq2 software package. Genes for which less than 20 reads were obtained from as a sum of all samples were excluded from the analysis. Of the 1000 most variable genes, 856 genes with an adjusted p-value for at least 2-fold differential expression between the wildtype and each of the three *roX* mutants exceeding 0.01 were also excluded from the analysis. In addition, the *white* gene and its upstream neighbors (*CG3588*, *CG14416*, *CG14417*, *CG14418* and *CG14419*) were excluded from the analysis due to strain background dissimilarities among strains in this genomic region. In total, 2356, 2659, 2571, 3164, 105, 10750 and 2042 genes on chromosomes 2L, 2R, 3L, 3R, 4, all autosomes except chromosome 4, and X, respectively, were included. For each of these genes, the average differential expression between replicates was log2-transformed and mean-centred, by subtracting the mean log2 fold change in expression of genes on the major autosomes (2L, 2R, 3L, 3R) from the value for each individual gene (S2 Table). Thus, the observed differences are relative and based on the assumption that overall expression of the four major autosomal arms is constant under all relevant conditions.

### Distance to High Affinity Sites (HAS) and Pioneer on the X sites (PionX)

The coordinates of PionX sites used in the analysis have been previously published [81], and the HAS coordinates on the X-chromosome were extracted from available data [45, 51], compiled and kindly provided by Philip and Stenberg [74]. The HAS coordinates were converted from release 5 to release 6 of the *Drosophila* genome using the flybase.org online conversion tool. The distances to the closest PionX and HAS sites were calculated for each gene on the X-chromosome, then genes were ranked in order of increasing distances to these sites and split into 10 bins with equal numbers of genes (S2 Table).

### MSL1, MSL3 or MOF binding bins

Binding values of MSL1, MSL3 and MOF in S2 cells were calculated and kindly provided by Philip and Stenberg [74] using the E-MEXP-1508 chromatin immunoprecipitation dataset [46] (S2 Table). Only X chromosomal genes with binding values for all three proteins were included in the analysis (1640 genes). Genes were ranked by increasing binding value and split into five equal bins. Genes located within MSL1 binding sites in the *roX1 roX2* mutant were determined using previously obtained ChIP data [14]. The percentage overlap between genes and the previously defined top 1.5% of peaks was calculated using the annotate function of BEDTools. A gene was considered to be within a MSL1 binding peak if any of its transcripts had at least 1% overlap.

### Classification of genes into early or late replicating

Bed files with data on early and late replicating domains in S2, Kc167 and DmBG3 cell lines were kindly provided by David MacAlpine [58]. The coordinates were converted from *Drosophila* genome release 5 to release 6 using flybase.org’s online coordinate converter. The annotate tool from BEDTools [82] was used to calculate the overlap between genes and replication domains. Genes were classified as early or late replicating in a given cell line if the entire transcript was within an early or late replicating domain.

### Chromatin immunoprecipitation and quantitative polymerase chain reaction analyses

Two replicates of formaldehyde cross-linked chromatin from third instar larvae of each strain were prepared according to a previously published protocol [83], then subjected to immunoprecipitation analysis with polyclonal rabbit anti-MSL1 antibodies, rabbit anti-H4K16ac antibodies (Millipore) or rabbit serum (mock negative control). Quantitative PCR was performed using SybrFast qPCR Master Mix (Kapa Biosystems), PCR primers listed in S1 Table, and a CFX Connect Real Time System (Bio-Rad laboratories).

### Defining testis-biased upregulated genes

Since the distribution of expression ratios varied between mutants we defined upregulated genes as those with a log2 fold change above the third quartile of the autosomal set (combined set of autosomes excluding chromosome 4). This resulted in thresholds for transcription up-regulation of 0.2, 0.068 and 0.307 for *roX1*, *roX2* and *roX1 rox2* mutant, respectively. The expression in testis data were extracted from the FlyAtlas2 database [84] (S2 Table). Gene with testis-enrichment values above 4 were classified as testis-biased.

### Bioinformatics and visualization

All calculations were performed using R [85] and plots were generated using the ggplot2 R package [86].

## Supporting information

S1 Table PCR primers used in the study. (PDF)

S2 Table Normalized RNA-seq dataset. (XLSX)

S3 Table Pair-wise Pearson correlation coefficients between all RNA-seq samples. (XLSX)

S1 Fig RNA *in situ* hybridization and immunostaining of male larval neuroblasts. **(A)** *roX1* RNA (yellow) and MLE protein (green) colocolize in mitotic and interphase nuclei. Arrows indicate the distal region of the X chromosome bound by both *roX1* and MLE. Examples of colocalization in X-territory of interphase nuclei are indicated by arrowheads. Five preparations were examined in total. **(B)** The binding of *roX1* RNA to the metaphase X chromosome is not redundant. In *roX1* mutant larval neuroblasts, the *roX2* RNA is observed in the X-territory of interphase nuclei (arrowheads), but not on the metaphase X chromosome. Ten preparations were examined in total.
(PDF)

S2 Fig Breakpoint sequences of the CRISPR-Cas9 generated new *roX2* mutants alleles. Sequences on the left and right sides of the deletions are highlighted in yellow and blue, respectively. Deletion sizes are shown in brackets.
(PDF)

S3 Fig Density plots of the average expression ratios for each chromosome arm. The average plot for 2L, 2R, 3L, 3R is denoted A. The vertical bar indicates 0.
(PDF)

S4 Fig Scatterplot of expression ratios versus distance to **(A)** high-affinity sites and **(B)** PionX sites. Each dot represents a single gene on the X-chromosome. Lowess fitting curve is shown by dashed line.
(PDF)

S5 Fig Average expression ratios of X chromosomal genes grouped in equal sized bins based on their binding strength (1 lowest to 5 highest). **(A-C)** show ratios based on MSL3-binding strength, **(D-F)** show ratios based on MOF-binding strength; **(A, D)** *roX1* –WT, **(B, E)** *roX2* –WT, **(C, F)** *roX1 roX2*—WT. The error bars represent 95% confidence intervals.
(PDF)

S6 Fig Differential effect of *roX* mutations on early and late replicating genes. Average expression ratios of X chromosomal (X) and autosomal (A) genes grouped by their replication time in **(A-C)** Kc167 cultured cells and **(D-F)** DmBG3 cultured cells. The expression ratios are calculated from the RNA-seq analysis on first instar larvae. The error bars represent 95% confidence intervals.
(PDF)

S7 Fig Upregulation of genes in the *roX2* mutant is not due to MSL complex re-distribution. ChIP-qPCR analysis of *roX2* mutant and wildtype 3^rd^ instar larvae, using antibody against **(A)** MSL1, **(B)** H4K16ac and **(C)** rabbit serum. Note the weak MSL1 and H4K16 signals from the weakly-expressed genes (*jb*, *whe*, *CG12679*, *CR32652*, *CG15306*, *TrxT*, *CG2574*, *CG11106*) in both fly strains in contrast to the strong signals from known MSL complex targets (*Gbeta13F*, *l(1)G0004*, *UbcE2H* and *roX1*). Genes *RpS3* and *Rpl32* are included as autosomal controls.
(PDF)

## References

[1] Mank JE. Sex chromosome dosage compensation: definitely not for everyone. Trends Genet. 2013;29: 677–683. 10.1016/j.tig.2013.07.005 23953923

[2] Stenberg P, Larsson J. Buffering and the evolution of chromosome-wide gene regulation. Chromosoma. 2011;120: 213–225. 10.1007/s00412-011-0319-8 21505791 PMC3098985

[3] Prestel M, Feller C, Becker PB. Dosage compensation and the global re-balancing of aneuploid genomes. Genome Biol. 2010;11: 216 10.1186/gb-2010-11-8-216 20804581 PMC2945780

[4] Deng X, Hiatt JB, Nguyen DK, Ercan S, Sturgill D, Hillier LW, Schlesinger F, Davis CA, Reinke VJ, Gingeras TR, et al Evidence for compensatory upregulation of expressed X-linked genes in mammals, *Caenorhabditis elegans* and *Drosophila melanogaster*. Nat Genet. 2011;43: 1179–1185. 10.1038/ng.948 22019781 PMC3576853

[5] Pollex T, Heard E. Recent advances in X-chromosome inactivation research. Curr Opin Cell Biol. 2012;24: 825–832. 10.1016/j.ceb.2012.10.007 23142477

[6] Lee JT. Epigenetic regulation by long noncoding RNAs. *Science*. 2012, 338: 1435–1439. 10.1126/science.1231776 23239728

[7] Brockdorff N. Noncoding RNA and Polycomb recruitment. RNA. 2013;19: 429–442. 10.1261/rna.037598.112 23431328 PMC3677253

[8] Stenberg P, Lundberg LE, Johansson AM, Rydén P, Svensson MJ, Larsson J. Buffering of segmental and chromosomal aneuploidies in *Drosophila melanogaster*. PLoS Genet. 2009;5: e100302. 10.1371/journal.pgen.1000465 PMC2668767 19412336

[9] Zhang Y, Malone JH, Powell SK, Periwal V, Spana E, MacAlpine DM, Oliver B. Expression in aneuploid *Drosophila* S2 cells. PLoS Biol. 2010;8: e1000320 10.1371/journal.pbio.1000320 20186269 PMC2826376

[10] Lundberg LE, Figueiredo ML, Stenberg P, Larsson J. Buffering and proteolysis are induced by segmental monosomy in *Drosophila melanogaster*. Nucleic Acids Res. 2012, 40: 5926–5937. 10.1093/nar/gks245 22434883 PMC3401434

[11] Gelbart ME, Kuroda MI. *Drosophila* dosage compensation: a complex voyage to the X chromosome. Development. 2009;136: 1399–1410. 10.1242/dev.029645 19363150 PMC2674252

[12] Conrad T, Akhtar A. Dosage compensation in *Drosophila melanogaster*: epigenetic fine-tuning of chromosome-wide transcription. Nat Rev Genet. 2011;13: 123–134. 10.1038/nrg3124 22251873

[13] Ilik IA, Quinn JJ, Georgiev P, Tavares-Cadete F, Maticzka D, Toscano S, Wan Y, Spitale RC, Luscombe N, Backofen R, et al Tandem stem-loops in roX RNAs act together to mediate X chromosome dosage compensation in *Drosophila*. Mol Cell. 2013;51: 156–173. 10.1016/j.molcel.2013.07.001 23870142 PMC3804161

[14] Figueiredo ML, Kim M, Philip P, Allgardsson A, Stenberg P, Larsson J. Non-coding *roX* RNAs prevent the binding of the MSL-complex to heterochromatic regions. PLoS Genet. 2014;10: e1004865 10.1371/journal.pgen.1004865 25501352 PMC4263465

[15] Ferrari F, Alekseyenko AA, Park PJ, Kuroda MI. Transcriptional control of a whole chromosome: emerging models for dosage compensation. Nat Struct Mol Biol. 2014;21: 118–125. 10.1038/nsmb.2763 24500429 PMC4342042

[16] Ferrari F, Jung YL, Kharchenko PV, Plachetka A, Alekseyenko AA, Kuroda MI, Park PJ. Comment on "*Drosophila* dosage compensation involves enhanced Pol II recruitment to male X-linked promoters". Science. 2013;340: 273 10.1126/science.1231815 23599463 PMC3665607

[17] Straub T, Becker PB. Comment on "*Drosophila* dosage compensation involves enhanced Pol II recruitment to male X-linked promoters". Science. 2013;340: 273 10.1126/science.1231895 23599464

[18] Conrad T, Cavalli FM, Vaquerizas JM, Luscombe NM, Akhtar A. *Drosophila* dosage compensation involves enhanced Pol II recruitment to male X-linked promoters. Science. 2012;337: 742–746. 10.1126/science.1221428 22821985

[19] Vaquerizas JM, Cavalli FM, Conrad T, Akhtar A, Luscombe NM. Response to Comments on "*Drosophila* dosage compensation involves enhanced Pol II recruitment to male X-linked promoters". Science. 2013;340: 273 10.1126/science.1232874 23599465

[20] Larschan E, Bishop EP, Kharchenko PV, Core LJ, Lis JT, Park PJ, Kuroda MI. X chromosome dosage compensation via enhanced transcriptional elongation in *Drosophila*. Nature. 2011;471: 115–118. 10.1038/nature09757 21368835 PMC3076316

[21] Prabhakaran M, Kelley RL. Mutations in the transcription elongation factor SPT5 disrupt a reporter for dosage compensation in *Drosophila*. PLoS Genet. 2012;8: e1003073 10.1371/journal.pgen.1003073 23209435 PMC3510053

[22] Sun L, Fernandez HR, Donohue RC, Li J, Cheng J, Birchler JA. Male-specific lethal complex in *Drosophila* counteracts histone acetylation and does not mediate dosage compensation. Proc Natl Acad Sci U S A. 2013;110: E808–817. 10.1073/pnas.1222542110 23382189 PMC3587201

[23] Franke A, Baker BS. The *rox1* and *rox2* RNAs are essential components of the Compensasome, which mediates dosage compensation in *Drosophila*. Mol Cell. 1999;4: 117–122. 10445033 10.1016/s1097-2765(00)80193-8

[24] Park SW, Kang YIe, Sypula JG, Choi J, Oh H, Park Y. An evolutionarily conserved domain of *roX2* RNA is sufficient for induction of H4-Lys16 acetylation on the *Drosophila* X chromosome. Genetics. 2007;177: 1429–1437. 10.1534/genetics.107.071001 18039876 PMC2147973

[25] Park SW, Kuroda MI, Park Y. Regulation of histone H4 Lys16 acetylation by predicted alternative secondary structures in *roX* noncoding RNAs. Mol Cell Biol. 2008;28: 4952–4962. 10.1128/MCB.00219-08 18541664 PMC2519712

[26] Kelley RL, Lee OK, Shim YK. Transcription rate of noncoding *roX1* RNA controls local spreading of the *Drosophila* MSL chromatin remodeling complex. Mech Dev. 2008;125: 1009–1019. 10.1016/j.mod.2008.08.003 18793722 PMC2659721

[27] Meller VH, Wu KH, Roman G, Kuroda MI, Davis RL. *roX1* RNA paints the X chromosome of male *Drosophila* and is regulated by the dosage compensation system. Cell. 1997;88: 445–457. 9038336 10.1016/s0092-8674(00)81885-1

[28] Meller VH, Gordadze PR, Park Y, Chu X, Stuckenholz C, Kelley RL, Kuroda MI. Ordered assembly of *roX* RNAs into MSL complexes on the dosage-compensated X chromosome in *Drosophila*. Curr Biol. 2000;10: 136–143. 10679323 10.1016/s0960-9822(00)00311-0

[29] Deng X, Rattner BP, Souter S, Meller VH. The severity of *roX1* mutations is predicted by MSL localization on the X chromosome. Mech Dev. 2005;122: 1094–1105. 10.1016/j.mod.2005.06.004 16125915

[30] Meller VH, Rattner BP. The *roX* genes encode redundant male-specific lethal transcripts required for targeting of the MSL complex. EMBO J. 2002;21: 1084–1091. 10.1093/emboj/21.5.1084 11867536 PMC125901

[31] Menon DU, Meller VH. Imprinting of the Y chromosome influences dosage compensation in *roX1 roX2 Drosophila melanogaster*. Genetics. 2009;183: 811–820. 10.1534/genetics.109.107219 19704014 PMC2778978

[32] Figueiredo ML, Philip P, Stenberg P, Larsson J. HP1a recruitment to promoters is independent of H3K9 methylation in *Drosophila melanogaster*. PLoS Genet. 2012;8: e1003061 10.1371/journal.pgen.1003061 23166515 PMC3499360

[33] Oh H, Park Y, Kuroda MI. Local spreading of MSL complexes from *roX* genes on the *Drosophila* X chromosome. Genes Dev. 2003;17: 1334–1339. 10.1101/gad.1082003 12782651 PMC196065

[34] Menon DU, Coarfa C, Xiao W, Gunaratne PH, Meller VH. siRNAs from an X-linked satellite repeat promote X-chromosome recognition in *Drosophila melanogaster*. Proc Natl Acad Sci U S A. 2014;111: 16460–16465. 10.1073/pnas.1410534111 25368194 PMC4246271

[35] Joshi SS, Meller VH. Satellite repeats identify X chromatin for dosage compensation in *Drosophila melanogaster* males. Curr Biol. 2017;27: 1393–1402 e1392. 10.1016/j.cub.2017.03.078 28457869 PMC5497753

[36] Menon DU, Meller VH. A role for siRNA in X-chromosome dosage compensation in *Drosophila melanogaster*. Genetics. 2012;191: 1023–1028. 10.1534/genetics.112.140236 22554892 PMC3389965

[37] Johansson AM, Allgardsson A, Stenberg P, Larsson J. *msl2* mRNA is bound by free nuclear MSL complex in *Drosophila melanogaster*. Nucleic Acids Res. 2011;39: 6428–6439. 10.1093/nar/gkr236 21551218 PMC3159442

[38] Straub T, Neumann MF, Prestel M, Kremmer E, Kaether C, Haass C, Becker PB. Stable chromosomal association of MSL2 defines a dosage-compensated nuclear compartment. Chromosoma. 2005;114: 352–364. 10.1007/s00412-005-0020-x 16179989

[39] Strukov YG, Sural TH, Kuroda MI, Sedat JW. Evidence of activity-specific, radial organization of mitotic chromosomes in *Drosophila*. PLoS Biol. 2011;9: e1000574 10.1371/journal.pbio.1000574 21264350 PMC3019107

[40] Deng X, Meller VH. *roX* RNAs are required for increased expression of X-linked genes in *Drosophila melanogaster* males. Genetics. 2006;174: 1859–1866. 10.1534/genetics.106.064568 17028315 PMC1698640

[41] Vicoso B, Bachtrog D. Reversal of an ancient sex chromosome to an autosome in *Drosophila*. Nature. 2013;499: 332–335. 10.1038/nature12235 23792562 PMC4120283

[42] Vicoso B, Bachtrog D. Numerous transitions of sex chromosomes in Diptera. PLoS Biol. 2015;13: e1002078 10.1371/journal.pbio.1002078 25879221 PMC4400102

[43] Deng X, Koya SK, Kong Y, Meller VH. Coordinated regulation of heterochromatic genes in *Drosophila melanogaster* males. Genetics. 2009;182: 481–491. 10.1534/genetics.109.102087 19307603 PMC2691757

[44] Hamada FN, Park PJ, Gordadze PR, Kuroda MI. Global regulation of X chromosomal genes by the MSL complex in *Drosophila melanogaster*. Genes Dev. 2005;19: 2289–2294. 10.1101/gad.1343705 16204180 PMC1240037

[45] Straub T, Grimaud C, Gilfillan GD, Mitterweger A, Becker PB. The chromosomal high-affinity binding sites for the *Drosophila* dosage compensation complex. PLoS Genet. 2008;4: e1000302 10.1371/journal.pgen.1000302 19079572 PMC2586088

[46] Kind J, Vaquerizas JM, Gebhardt P, Gentzel M, Luscombe NM, Bertone P, Akhtar A. Genome-wide analysis reveals MOF as a key regulator of dosage compensation and gene expression in *Drosophila*. Cell. 2008;133: 813–828. 10.1016/j.cell.2008.04.036 18510926

[47] Lyman LM, Copps K, Rastelli L, Kelley RL, Kuroda MI. *Drosophila* male-specific lethal-2 protein: structure/function analysis and dependence on MSL-1 for chromosome association. Genetics. 1997;147: 1743–1753. 9409833 10.1093/genetics/147.4.1743 PMC1208343

[48] Gu W, Szauter P, Lucchesi JC. Targeting of MOF, a putative histone acetyl transferase, to the X chromosome of *Drosophila melanogaster*. Dev Genet. 1998;22: 56–64. 10.1002/(SICI)1520-6408(1998)22:1<56::AID-DVG6>3.0.CO;2-6 9499580

[49] Demakova OV, Kotlikova IV, Gordadze PR, Alekseyenko AA, Kuroda MI, Zhimulev IF. The MSL complex levels are critical for its correct targeting to the chromosomes in *Drosophila melanogaster*. Chromosoma. 2003;112: 103–115. 10.1007/s00412-003-0249-1 14579126

[50] Dahlsveen IK, Gilfillan GD, Shelest VI, Lamm R, Becker PB. Targeting determinants of dosage compensation in *Drosophila*. PLoS Genet. 2006;2: e5 10.1371/journal.pgen.0020005 16462942 PMC1359073

[51] Alekseyenko AA, Peng S, Larschan E, Gorchakov AA, Lee OK, Kharchenko P, McGrath SD, Wang CI, Mardis ER, Park PJ, Kuroda MI. A sequence motif within chromatin entry sites directs MSL establishment on the *Drosophila* X chromosome. Cell. 2008;134: 599–609. 10.1016/j.cell.2008.06.033 18724933 PMC2613042

[52] Straub T, Zabel A, Gilfillan GD, Feller C, Becker PB. Different chromatin interfaces of the *Drosophila* dosage compensation complex revealed by high-shear ChIP-seq. Genome Res. 2013;23: 473–485. 10.1101/gr.146407.112 23233545 PMC3589536

[53] Rivera-Mulia JC, Gilbert DM. Replication timing and transcriptional control: beyond cause and effect-part III. Curr Opin Cell Biol. 2016;40: 168–178. 10.1016/j.ceb.2016.03.022 27115331 PMC4887323

[54] Fraser HB. Cell-cycle regulated transcription associates with DNA replication timing in yeast and human. Genome Biol. 2013;14: R111 10.1186/gb-2013-14-10-r111 24098959 PMC3983658

[55] Hatton KS, Dhar V, Brown EH, Iqbal MA, Stuart S, Didamo VT, Schildkraut CL. Replication program of active and inactive multigene families in mammalian cells. Mol Cell Biol. 1988;8: 2149–2158. 3386634 10.1128/mcb.8.5.2149-2158.1988 PMC363396

[56] Schubeler D, Scalzo D, Kooperberg C, van Steensel B, Delrow J, Groudine M. Genome-wide DNA replication profile for *Drosophila melanogaster*: a link between transcription and replication timing. Nat Genet. 2002;32: 438–442. 10.1038/ng1005 12355067

[57] Stambrook PJ, Flickinger RA. Changes in chromosomal DNA replication patterns in developing frog embryos. J Exp Zool. 1970;174: 101–113. 10.1002/jez.1401740110 5444565

[58] Lubelsky Y, Prinz JA, DeNapoli L, Li Y, Belsky JA, MacAlpine DM. DNA replication and transcription programs respond to the same chromatin cues. Genome Res. 2014;24: 1102–1114. 10.1101/gr.160010.113 24985913 PMC4079966

[59] Dimitrova DS, Gilbert DM. The spatial position and replication timing of chromosomal domains are both established in early G1 phase. Mol Cell. 1999;4: 983–993. 10635323 10.1016/s1097-2765(00)80227-0

[60] Quinn JJ, Zhang QC, Georgiev P, Ilik IA, Akhtar A, Chang HY. Rapid evolutionary turnover underlies conserved lncRNA-genome interactions. Genes Dev. 2016;30: 191–207. 10.1101/gad.272187.115 26773003 PMC4719309

[61] Nowak MA, Boerlijst MC, Cooke J, Smith JM. Evolution of genetic redundancy. Nature. 1997;388: 167–171. 10.1038/40618 9217155

[62] Belote JM, Lucchesi JC. Male-specific lethal mutations of *Drosophila melanogaster*. Genetics. 1980;96: 165–186. 6781985 10.1093/genetics/96.1.165 PMC1214287

[63] Hochman B. The fourth chromosome of *Drosophila melanogaster* In Ashburner M, Novitski E, editors. The Genetics and biology of *Drosophila*. Volume 1B Academic Press; 1976 pp. 903–928

[64] Johansson AM, Stenberg P, Bernhardsson C, Larsson J. Painting of fourth and chromosome-wide regulation of the 4th chromosome in *Drosophila melanogaster*. EMBO J. 2007;26: 2307–2316. 10.1038/sj.emboj.7601604 17318176 PMC1864965

[65] Johansson AM, Stenberg P, Pettersson F, Larsson J. POF and HP1 bind expressed exons, suggesting a balancing mechanism for gene regulation. PLoS Genet. 2007;3: e209 10.1371/journal.pgen.0030209 18020713 PMC2077892

[66] Johansson AM, Stenberg P, Allgardsson A, Larsson J. POF regulates the expression of genes on the fourth chromosome in *Drosophila melanogaster* by binding to nascent RNA. Mol Cell Biol. 2012;32: 2121–2134. 10.1128/MCB.06622-11 22473994 PMC3372238

[67] Chen ZX, Oliver B. X chromosome and autosome dosage responses in *Drosophila melanogaster* heads. G3 (Bethesda). 2015;5: 1057–1063. 10.1534/g3.115.017632 25850426 PMC4478536

[68] Bachtrog D, Toda NR, Lockton S. Dosage compensation and demasculinization of X chromosomes in *Drosophila*. Curr Biol. 2010;20: 1476–1481. 10.1016/j.cub.2010.06.076 20705467 PMC4511158

[69] Larsson J, Chen JD, Rasheva V, Rasmuson Lestander A, Pirrotta V. Painting of fourth, a chromosome-specific protein in *Drosophila*. Proc Natl Acad Sci U S A. 2001;98: 6273–6278. 10.1073/pnas.111581298 11353870 PMC33458

[70] Larsson J, Svensson MJ, Stenberg P, Mäkitalo M. Painting of fourth in genus *Drosophila* suggests autosome-specific gene regulation. Proc Natl Acad Sci U S A. 2004;101: 9728–9733. 10.1073/pnas.0400978101 15210994 PMC470743

[71] Kim M, Ekhteraei-Tousi S, Lewerentz J, Larsson J. The X-linked 1.688 satellite in *Drosophila melanogaster* promotes specific targeting by Painting of Fourth. Genetics. 2018;208: 623–632. 10.1534/genetics.117.300581 29242291 PMC5788526

[72] Gupta V, Parisi M, Sturgill D, Nuttall R, Doctolero M, Dudko OK, Malley JD, Eastman PS, Oliver B. Global analysis of X-chromosome dosage compensation. J Biol. 2006;5: 3 10.1186/jbiol30 16507155 PMC1414069

[73] Sturgill D, Zhang Y, Parisi M, Oliver B. Demasculinization of X chromosomes in the *Drosophila* genus. Nature. 2007;450: 238–241. 10.1038/nature06330 17994090 PMC2386140

[74] Philip P, Stenberg P. Male X-linked genes in *Drosophila melanogaster* are compensated independently of the Male-Specific Lethal complex. Epigenetics Chromatin. 2013;6: 35 10.1186/1756-8935-6-35 24279328 PMC4176495

[75] Ellison CE, Bachtrog D. Dosage compensation via transposable element mediated rewiring of a regulatory network. Science. 2013;342: 846–850. 10.1126/science.1239552 24233721 PMC4086361

[76] Alekseyenko AA, Ellison CE, Gorchakov AA, Zhou Q, Kaiser VB, Toda N, Walton Z, Peng S, Park PJ, Bachtrog D, Kuroda MI. Conservation and *de novo* acquisition of dosage compensation on newly evolved sex chromosomes in *Drosophila*. Genes Dev. 2013;27: 853–858. 10.1101/gad.215426.113 23630075 PMC3650223

[77] Deng X, Meller VH. Molecularly severe *roX1* mutations contribute to dosage compensation in *Drosophila*. Genesis. 2009;47: 49–54. 10.1002/dvg.20463 19101984 PMC5029428

[78] Kondo S, Ueda R. Highly improved gene targeting by germline-specific Cas9 expression in *Drosophila*. Genetics. 2013;195: 715–721. 10.1534/genetics.113.156737 24002648 PMC3813859

[79] Lundberg LE, Kim M, Johansson AM, Faucillion ML, Josupeit R, Larsson J. Targeting of Painting of fourth to *roX1* and *roX2* proximal sites suggests evolutionary links between dosage compensation and the regulation of the fourth chromosome in *Drosophila melanogaster*. G3 (Bethesda). 2013;3: 1325–1334. 10.1534/g3.113.006866 23733888 PMC3737172

[80] Sullivan W, Ashburner M, Hawley RS. *Drosophila* protocols. Cold Spring Harbor, New York: Cold Spring Harbor Laboratory Press; 2000.

[81] Villa R, Schauer T, Smialowski P, Straub T, Becker PB. PionX sites mark the X chromosome for dosage compensation. Nature. 2016;537: 244–248. 10.1038/nature19338 27580037

[82] Quinlan AR, Hall IM. BEDTools: a flexible suite of utilities for comparing genomic features. Bioinformatics. 2010;26: 841–842. 10.1093/bioinformatics/btq033 20110278 PMC2832824

[83] Kahn TG, Dorafshan E, Schultheis D, Zare A, Stenberg P, Reim I, Pirrotta V, Schwartz YB. Interdependence of PRC1 and PRC2 for recruitment to Polycomb Response Elements. Nucleic Acids Res. 2016;44: 10132–10149. 10.1093/nar/gkw701 27557709 PMC5137424

[84] Leader DP, Krause SA, Pandit A, Davies SA, Dow JAT. FlyAtlas 2: a new version of the *Drosophila melanogaster* expression atlas with RNA-Seq, miRNA-Seq and sex-specific data. Nucleic Acids Res. 2018;46: D809–D815. 10.1093/nar/gkx976 29069479 PMC5753349

[85] R: A language and environment for statistical computing. Available from: https://www.r-project.org/

[86] Wickham H. ggplot2: Elegant graphics for data analysis. New York: Springer-Verlag; 2009.

