# Supplementary material for: RNA-on-X 1 and 2 in Drosophila melanogaster fulfill separate functions in dosage compensation
Source: PLoS Genet. 2018 Dec 10;14(12):e1007842. doi: 10.1371/journal.pgen.1007842 (PMC6301720; doi:10.1371/journal.pgen.1007842)
Supplement: S1 Table — (PDF) [file pgen.1007842.s001.pdf]

S1 Table. PCR primers

Amplified DNA segment	***Primer sequence***
jb	gctggcaggaatggactcga
	gctcatttaggcgctcgtgc
whe	catcgttgtgctcgccatgg
	gagccctgaagtggtcccag
CG12679	gctgatggacctcgctctgt
	ggcagccacaccgaagtaga
CR32652	ttactggatcgcatcgcctg
	gggaccggaagtgtgcagta
CG15306	cgcttggtgaaggtgaagacg
	tcctggttagctttgcccgt
TrxT	gcagcagtactcggaccgt
	gccgcccttgatgaacacaa
CG2574	ccagtcacagtgcatcggga
	gtgcaattcggtggcggatt
CG11106	tgtcctcaattccgggagcc
	ctccagatccaccgccgtag
Gbeta13F	gtccatgccattccactgcg
	cacatgttgtcgaggccacc
l(1)G0004	aacctaatcctcggctcgcc
	gctctcccaatcacatgcgc
UbcE2H	aggtggccgactatgtgcag
	ccctggcctcatcctcactg

roX1_1	acgaggtatggcatttccct
	tggccaccttatagagataaccc
roX1_2	ctttgggattgctgcacgat
	gacaagtggcagccctaatg
RpS3	cattgagttgtacgccgaga
	gaatggatcatcaggccatc
RpL32	cgatgttgggcatcagatac
	cccaagatcgtgaagaagc
roX2 genomic locus	gccgctggaggcaaggatatgag
	cagaactctgttaatgcg
