# Supplementary figures and images for: RNA-on-X 1 and 2 in Drosophila melanogaster fulfill separate functions in dosage compensation
Source: PLoS Genet. 2018 Dec 10;14(12):e1007842. doi: 10.1371/journal.pgen.1007842 (PMC6301720; doi:10.1371/journal.pgen.1007842)

S1 fig

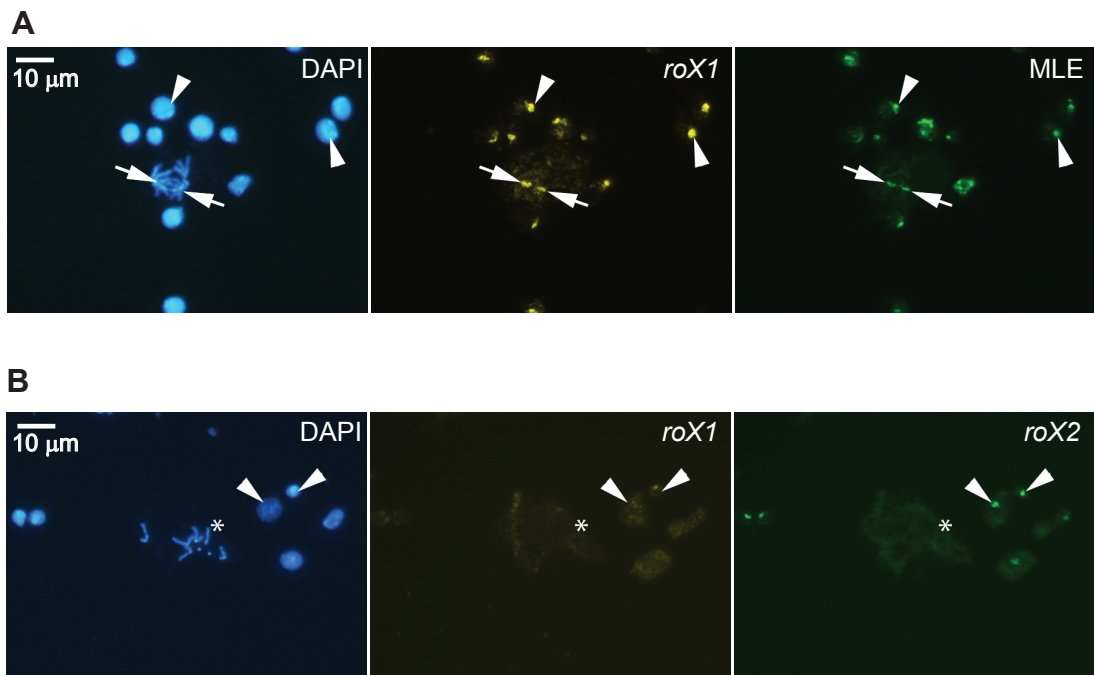

Supplement: S1 Fig — (A) roX1 RNA (yellow) and MLE protein (green) colocolize in mitotic and interphase nuclei. Arrows indicate the distal region of the X chromosome bound by both roX1 and MLE. Examples of colocalization in X-territory of interphase nuclei are indicated by arrowheads. Five preparations were examined in total. (B) The binding of roX1 RNA to the metaphase X chromosome is not redundant. In roX1 mutant larval neuroblasts, the roX2 RNA is observed in the X-territory of interphase nuclei (arrowheads), but not on the metaphase X chromosome. Ten preparations were examined in total. (PDF) [file pgen.1007842.s004.pdf]

S3 fig

Density of gene expression log2 fold change

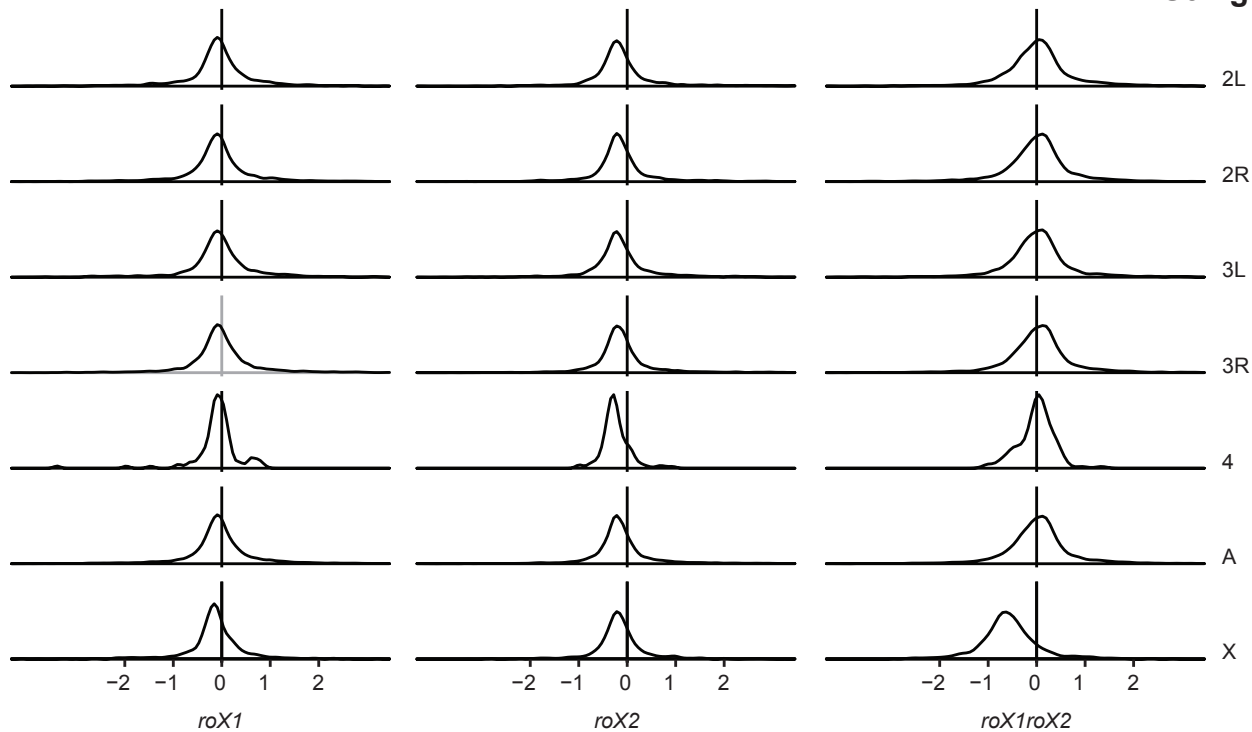

Supplement: S3 Fig — The average plot for 2L, 2R, 3L, 3R is denoted A. The vertical bar indicates 0. (PDF) [file pgen.1007842.s006.pdf]

S4 fig

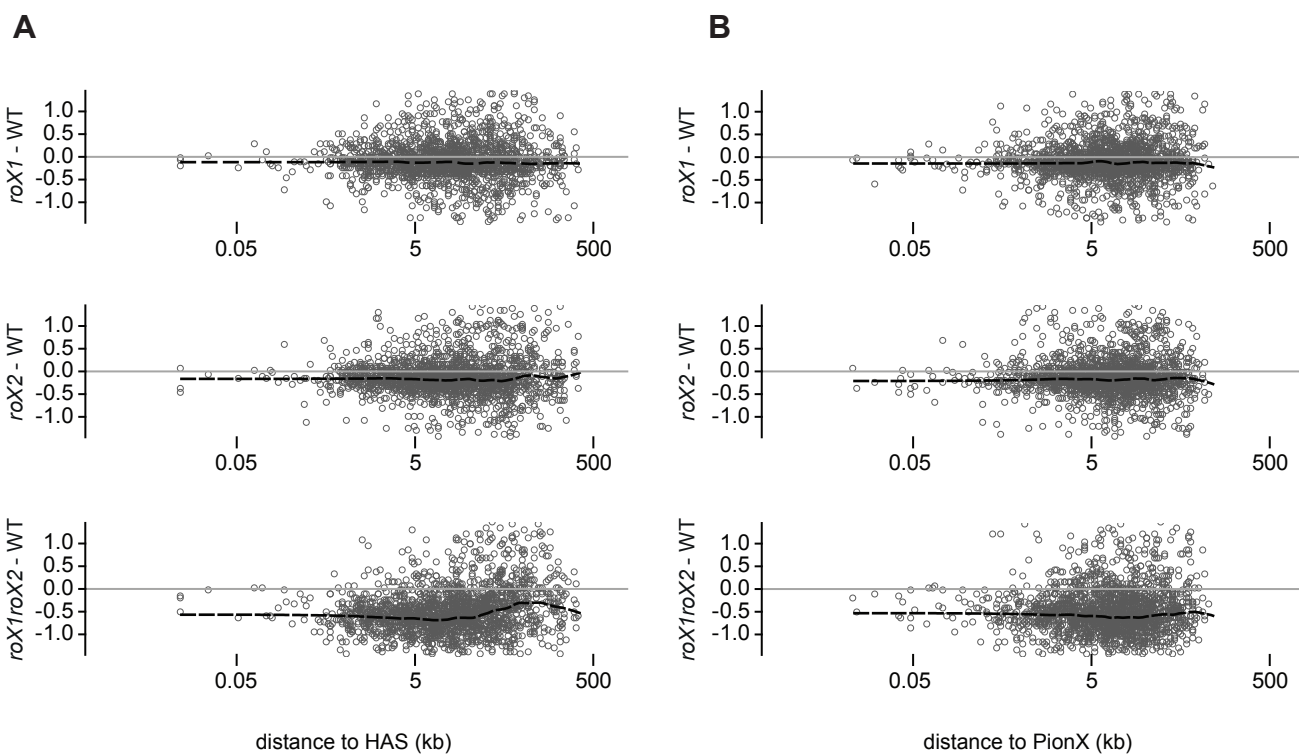

Supplement: S4 Fig — Scatterplot of expression ratios versus distance to (A) high-affinity sites and (B) PionX sites. Each dot represents a single gene on the X-chromosome. Lowess fitting curve is shown by dashed line. (PDF) [file pgen.1007842.s007.pdf]

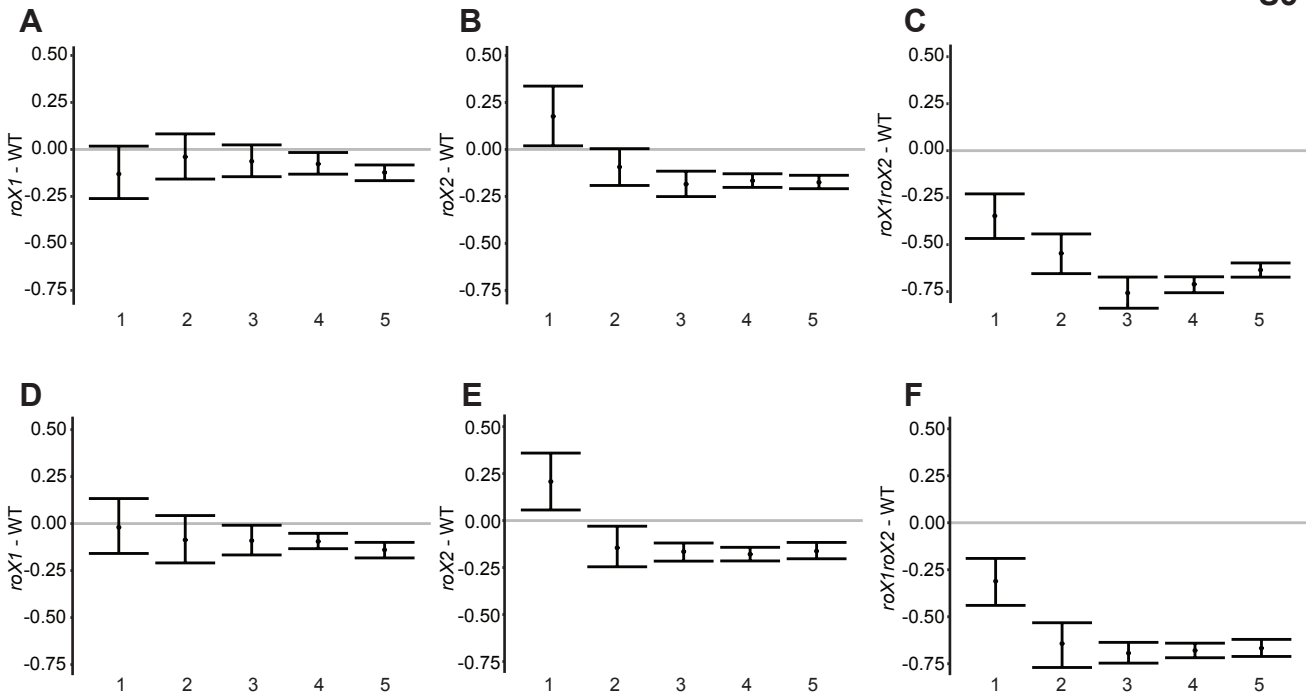

Supplement: S5 Fig — (A-C) show ratios based on MSL3-binding strength, (D-F) show ratios based on MOF-binding strength; (A, D) roX1 –WT, (B, E) roX2 –WT, (C, F) roX1 roX2—WT. The error bars represent 95% confidence intervals. (PDF) [file pgen.1007842.s008.pdf]

**A**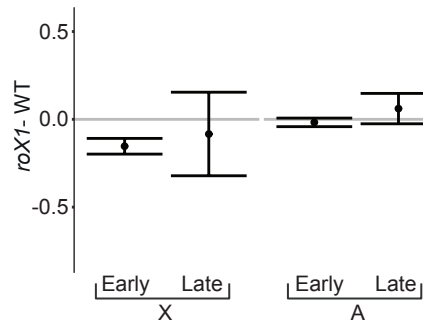**B**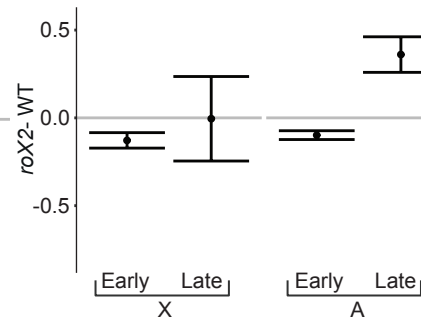**C**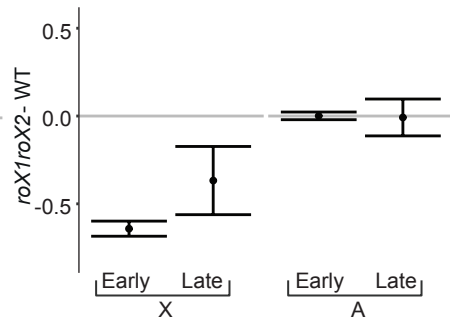**D**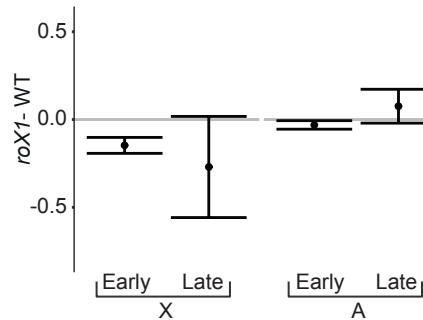**E**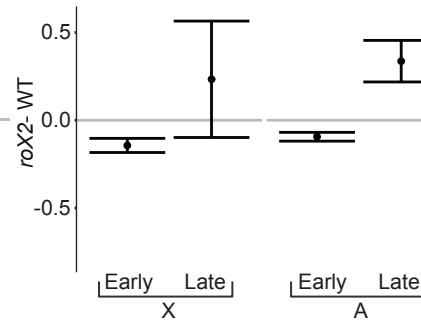**F**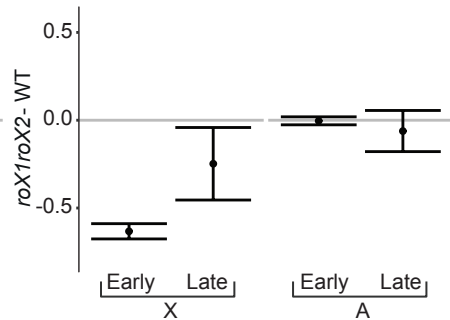

Supplement: S6 Fig — Average expression ratios of X chromosomal (X) and autosomal (A) genes grouped by their replication time in (A-C) Kc167 cultured cells and (D-F) DmBG3 cultured cells. The expression ratios are calculated from the RNA-seq analysis on first instar larvae. The error bars represent 95% confidence intervals. (PDF) [file pgen.1007842.s009.pdf]

**S7 fig**

■ roX2 replicate 1   ■ roX2 replicate 2   ■ wt replicate 1   ■ wt replicate 2

**A**

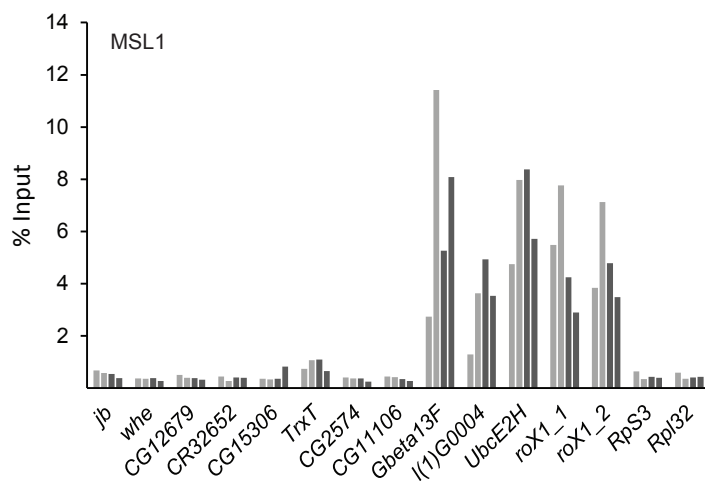

**B**

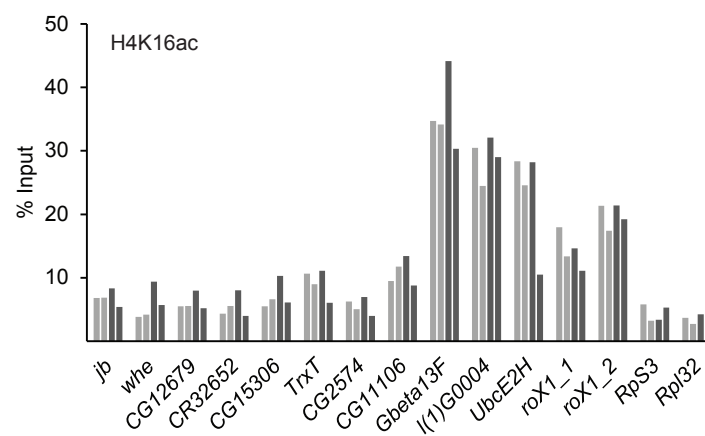

**C**

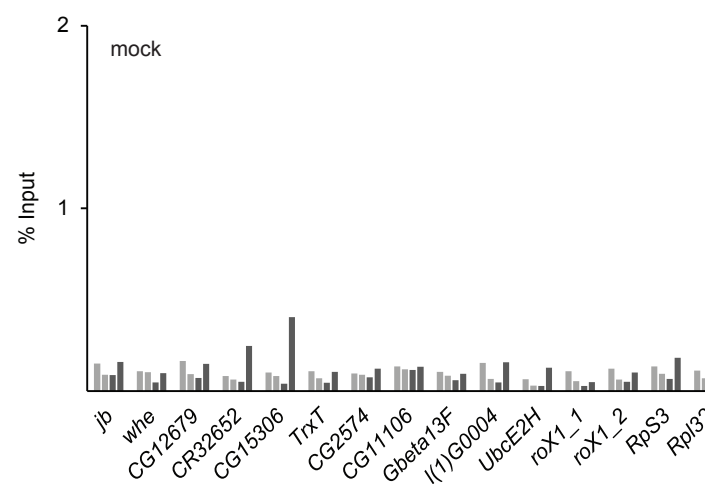

Supplement: S7 Fig — ChIP-qPCR analysis of roX2 mutant and wildtype 3rd instar larvae, using antibody against (A) MSL1, (B) H4K16ac and (C) rabbit serum. Note the weak MSL1 and H4K16 signals from the weakly-expressed genes (jb, whe, CG12679, CR32652, CG15306, TrxT, CG2574, CG11106) in both fly strains in contrast to the strong signals from known MSL complex targets (Gbeta13F, l(1)G0004, UbcE2H and roX1). Genes RpS3 and Rpl32 are included as autosomal controls. (PDF) [file pgen.1007842.s010.pdf]
