# Supplementary material for: RNA-on-X 1 and 2 in Drosophila melanogaster fulfill separate functions in dosage compensation
Source: PLoS Genet. 2018 Dec 10;14(12):e1007842. doi: 10.1371/journal.pgen.1007842 (PMC6301720; doi:10.1371/journal.pgen.1007842)
Supplement: S2 Fig — Sequences on the left and right sides of the deletions are highlighted in yellow and blue, respectively. Deletion sizes are shown in brackets. (PDF) [file pgen.1007842.s005.pdf]

S2 fig

4-2
ACATAAAACACATTTAATTCGAAATAAGTTTGGGTATTATTAGCTTATAATATTCAAAAGTGTATTTT
GCAATTGGAACGAATTTGGAATCCCGCTATTTTCGGATTCATGCAGTTCCCATTATATTTTATTCGGT
ATTGGAATCCCGCTATTTTCGCATTCATGCAGTTCCCACTATATTTTATTCGGTATTTTCGCAGTCAT
TAGTGTGGCCAAAACTCGAAAATATCAAGGGCTAGAGCAGCTAGATGTTGCGGCATTCGCGGCCTGGT
CACACTAAGCTAGGGCTACTTTTTATATCATAAGTCGAGCGTTTAGGTAAGCGAAACAAAAAGAGCTT
TAGTTAGAGGTATTAGTTTGGTTGCTATTATTTCTAAATTGAAAACTTCACTTCCTTTGATCCAAAAG
ACACTGAAAAGACACGTTTGCAGTTGAGTTCATTATTTTCTGGTATACATACACACTGATTACTCATT
CAATTGGCATTTTGCTCTTGTTTTTCTCCGATTGCCTTGCACTCGCATAAATTTAACACAAAAAAGAA
GTTCGGGGTGTTTAGAATCCATCCACTTGGTACAGTTCCCATCGAGCTGGTGAGTACTCCGCGCAGTG
CAACGTATACACACTTGAACTCGAATTCTGGCAGCAAAATGGTGGTGACCTATTAATAACACCCGCTC
AATTTTC-[Δ876bp]-TCCAGGGTGACCAGAACTGACACTTAAATTACATATGACAAATAAAGACTT
ATCTGCTATTGCAAATACTGTTTTAAGCGGCTTTTTTTCCATCAAATTTGTTAGTTCTTGTTCCTGTT
TTAATAAGTTGGCAATACCGATCCTCCGCACTTGCTATGTGTGTCTATAATCATATAGGGTGGCAGCG
CAGGCCAGGGCTGGCAAGGGTGGCGCATTAACAGAGTTCTG

8-5
ACATAAAACACATTTAATTCGAAATAAGTTTGGGTATTATTAGCTTATAATATTCAAAAGTGTATTTT
GCAATTGGAACGAATTTGGAATCCCGCTATTTTCGGATTCATGCAGTTCCCATTATATTTTATTCGGT
ATTGGAATCCCGCTATTTTCGCATTCATGCAGTTCCCACTATATTTTATTCGGTATTTTCGCAGTCAT
TAGTGT-[Δ1416bp]-AGCGCAGGCCAGGGCTGGCAAGGGTGGCGCATTAACAGAGTTCTG

9-4
ACATAAAACACATTTAATTCGAAATAAGTTTGGGTATTATTAGCTTATAATATTCAAAAGTGTATTTT
GCAATTGGAACGAATTTGGAATCCCGCTATTTTCGGATTCATGCAGTTCCCATTATATTTTATTCGGT
ATTGGAATCCCGCTATTTTCGCATTCATGCAGTTCCCACTATATTTTATTCGGTATTTTCGCAGTCAT
TAGTGTGGCCAAAACTCGAAAATATCAAGGGCTAGAGCAGCTAGAT-[Δ1313bp]-TCCAGGGTGAC
CAGAACTGACACTTAAATTACATATGACAAATAAAGACTTATCTGCTATTGCAAATACTGTTTTAAGC
GGCTTTTTTTCCATCAAATTTGTTAGTTCTTGTTCCTGTTTTAATAAGTTGGCAATACCGATCCTCCG
CACTTGCTATGTGTGTCTATAATCATATAGGGTGGCAGCGCAGGCCAGGGCTGGCAAGGGTGGCGCAT
TAACAGAGTTCTG

15-6
ACATAAAACACA-[Δ1554bp]-AGGGTGACCAGAACTGACACTTAAATTACATATGACAAATAAAGA
CTTATCTGCTATTGCAAATACTGTTTTAAGCGGCTTTTTTTCCATCAAATTTGTTAGTTCTTGTTCCT
GTTTTAATAAGTTGGCAATACCGATCCTCCGCACTTGCTATGTGTGTCTATAATCATATAGGGTGGCA
GCGCAGGCCAGGGCTGGCAAGGGTGGCGCATTAACAGAGTTCTG
